# Supplementary material for: Understanding the diversity of the metal-organic framework ecosystem
Source: Nat Commun. 2020 Aug 13;11:4068. doi: 10.1038/s41467-020-17755-8 (PMC7426948; doi:10.1038/s41467-020-17755-8)
Supplement: Supplementary file 1 — Supplementary Information [file 41467_2020_17755_MOESM1_ESM.pdf]

Supporting Information

Understanding the diversity of the metal-organic framework ecosystem

Moosavi et al.

Supplementary Note 1: Extended Details for the Material Featurisation 3
Supplementary Note 2: Metal-Organic Framework Databases 5
Supplementary Note 3: Statistics and Parties of ML Models 6
Supplementary Note 4: Partial Atomic Charges 13
Supplementary Note 5: Importance of Variables for Gas Adsorption Properties 16
Supplementary Note 6: PCA/TSNEs 21
Supplementary Note 7: Mining Metal Nodes from CoRE-MOF Database 25
Supplementary Note 8: Notes on KRR Models..27
Supplementary Note 9: Notes on RF and GBR models 29
Supplementary Note 10: Structure Refining Steps 30
Supplementary Note 11: Molecular Simulation Parameter Sets 32
Supplementary Note 12: Details of the Partial Charge Calculation using the EQeq method 33
Supplementary Note 13: Including hMOF in the Design Space 34
Supplementary Note 14: Metal Type Variation in Databases 38
Supplementary Note 15: Distribution of Adsorption Properties of Databases 39
Supplementary Note 16: Diversity Metrics 41
Supplementary Note 17: Learning Curves for Different Train Sets 47
Supplementary References 51

Supplementary Note 1: Extended Details for the Material Featurisation

The full feature set used in this study includes 165 descriptors for different domains of a MOF structure, namely geometric features of the pore, RACs chemical descriptors for metal chemistry, linker chemistry, and functional groups, and the cell volume.

Supplementary Table 1. Full list of the descriptors used in this study.

Material domain	notes	#descriptors
Geometry	-	8
Metal chemistry	Metal Center RACs	40
Linker chemistry	Linker Connecting and Full Linker RACs	68
Functional groups chemistry	Functional group RACs	48
Others	Cell Volume	1
		Total: 165

The geometric descriptors are largest included sphere (Di), largest free sphere (Df), largest included sphere along free path (Dif), crystal density (ρ), volumetric surface area (VSA), gravimetric surface (GSA), volumetric pore volume (VPOV) and gravimetric pore volume (GPOV). Also, we include cell volume as a descriptor.

To compute the RACs features, we use Supplementary Supplementary Equation (1) for the product RACs and equation 1 from the main text for difference RACs.

$$ {}_{\text{scope}}^{\text{start}}P_d^{\text{Prod}} = \sum_i^{\text{start}} \sum_j^{\text{scope}} P_i \, P_j \delta(d_{i,j}, d) \qquad \text{Supplementary Equation (1)} $$

The list of start and scope atom lists used in our study are shown in Supplementary Table2.

Supplementary Table 2. Details of the RACs descriptors used in this study to describe MOF chemistry.

RACs type	*Start*	*Scope*	No. Atomic properties	Max. Depth	#descriptors
Product	Full linker	Same Linker	5	3	20
	Linker connecting	Same Linker	6	3	24
	Metal center	Full MOF	5	3	20
	Functional group	Same Linker	6	3	24
Difference	Linker connecting	Same Linker	6	3	24
	Metal center	Full MOF	5	3	20
	Functional group	Same Linker	6	3	24
					Total: 156

To compute RACs, we start from the MOF crystal structure. We use pymatgen[1] to convert the crystal to its primitive cell.[2] The periodic pairwise distance matrix between all the atoms in the primitive cell is computed. The adjacency matrix is computed based on this pairwise distance matrix. Two atoms assigned to be bonded if their pairwise distance times a tuning factor is below the sum of their covalent radii (Supplementary Supplementary Equation (S 2)).

$$f \times r_{i,j} < (R_{cov,i} + R_{cov,j}) \qquad \text{Supplementary Equation (S 2)}$$

The tuning factor is 0.9 for most cases except for the bonds between metals and organic atoms that we tune this factor slightly depending on the atom types.[2] We do not allow metal-metal bonds. However, this tuning is not perfect and can lead to the incorrect adjacency matrix, specifically, in cases were the geometry of the atoms are not fully correct from the experimental crystal structure. We consider main group, alkali, alkaline earth, transition, metalloids, lanthanides, and actinides as metal in this study.[3]

Supplementary Note 2: Metal-Organic Framework Databases

Supplementary Table 3. The list of the databases investigated in this study.

Name used in this manuscript	*Type*	*Number of structures*	*Notes and references*
CoRE-2019	Experimental	~12,000	Computational Ready, Experimental MOFs initially developed[4] and later extended[5] by Chung et al.
CoRE-DDEC	Experimental	~3,000	The refined subset of CoRE-MOF database with DDEC partial charges developed by Nazarian et al.[6]
hMOF	Hypothetical	~130,000	Hypothetical MOFs generated by Wilmer et al.[7]
BW-DB	Hypothetical	~300,000	Hypothetical MOFs generated by Boyd et al.[8]
BW-20K	Hypothetical	~20,000	A diverse subset of structures from BW-DB
ToBaCCo	Hypothetical	~13,000	Hypothetical MOFs generated by Gomez-Gualdron et al.[9]
ARABG-DB	Hypothetical	~400	Hypothetical MOFs generated by Anderson et al.[10]

Supplementary Note 3: Statistics and Parties of ML Models

Supplementary Table 4. Accuracy of kernel ridge regression (KRR) models in prediction of gas adsorption properties of **CoRE-2019**. The machine learning models were trained using ~7,000 training data randomly chosen, and the statistics are reported for the remaining structures as the test set (~2,500 structures). All numbers were averaged over 10 different train-test splitting of the data. Units are similar to the main text. Henry coefficient (k_H), gas uptakes and deliverable capacity for CH_4, and gas uptakes for CO_2 are reported in $mol.kg^{-1}.Pa^{-1}$, vSTP/v, and $mol.kg^{-1}$, respectively. MAE: mean absolute error; RMAE: relative mean absolute error (%), and SRCC: Spearman ranking correlation coefficient.

	Property	Geo. Descriptors				Geo. & Chem. Descriptors			
		MAE	RMAE (%)	RMSE	SRCC	MAE	RMAE (%)	RMSE	SRCC
CH_4	$\log(k_H)$	0.29	4.77	0.43	0.67	0.20	3.26	0.35	0.84
	Upt@5.8 bar	19.59	7.34	27.15	0.75	12.94	4.85	19.41	0.88
	Upt@65 bar	19.94	5.36	27.26	0.92	16.64	4.47	25.02	0.94
	Del. Cap.	14.78	5.15	20.28	0.90	13.71	4.78	19.35	0.91
CO_2	$\log(k_H)$	0.74	8.29	0.98	0.50	0.51	5.64	0.74	0.77
	Upt@0.15 bar	0.92	9.90	1.28	0.57	0.57	6.12	0.85	0.81
	Upt@16 bar	0.97	2.87	1.51	0.95	0.97	2.87	1.51	0.95
Charges	MPC	0.28	10.15	0.39	0.44	0.07	2.54	0.16	0.93
	MNC	0.17	5.98	0.26	0.30	0.11	3.97	0.20	0.75

Supplementary Table 5. Accuracy of random forest regression (RF) models in prediction of gas adsorption properties of **CoRE-2019**. The machine learning models were trained using ~7,000 training data randomly chosen, and the statistics are reported for the remaining structures as the test set (~2,500 structures). All numbers were averaged over 10 different train-test splitting of the data. Units are similar to the main text. Henry coefficient (k_H), gas uptakes and deliverable capacity for CH_4, and gas uptakes for CO_2 are reported in $mol.kg^{-1}.Pa^{-1}$, vSTP/v, and $mol.kg^{-1}$, respectively. MAE: mean absolute error; RMAE: relative mean absolute error (%), and SRCC: Spearman ranking correlation coefficient.

	Property	Geo. Descriptors				Geo. & Chem. Descriptors			
		MAE	RMAE (%)	RMSE	SRCC	MAE	RMAE (%)	RMSE	SRCC
CH_4	$\log(k_H)$	0.26	4.19	0.38	0.71	0.16	2.70	0.26	0.87
	Upt@5.8 bar	17.83	6.68	25.69	0.77	12.70	4.76	19.00	0.88
	Upt@65 bar	18.03	4.84	25.65	0.92	14.05	3.78	20.53	0.95
	Del. Cap.	13.23	4.61	18.81	0.91	10.95	3.81	15.92	0.94
CO_2	$\log(k_H)$	0.66	7.40	0.91	0.59	0.42	4.63	0.63	0.83
	Upt@0.15 bar	0.89	9.54	1.23	0.60	0.56	6.05	0.85	0.82
	Upt@16 bar	0.83	2.46	1.30	0.96	0.65	1.92	1.05	0.97
Charges	MPC	0.26	9.09	0.36	0.51	0.06	2.06	0.13	0.95
	MNC	0.17	5.79	0.25	0.35	0.10	3.35	0.19	0.80

Supplementary Table 6. Accuracy of gradient boosting regression (GBR) models in prediction of gas adsorption properties of **CoRE-2019**. The machine learning models were trained using ~7,000 training data randomly chosen, and the statistics are reported for the remaining structures as the test set (~2,500 structures). All numbers were averaged over 10 different train-test splitting of the data. Units are similar to the main text. Henry coefficient (k_H), gas uptakes and deliverable capacity for CH_4, and gas uptakes for CO_2 are reported in mol.kg^{-1}.Pa^{-1}, vSTP/v, and mol.kg^{-1}, respectively. MAE: mean absolute error; RMAE: relative mean absolute error (%), and SRCC: Spearman ranking correlation coefficient.

	Property	Geo. Descriptors				Geo. & Chem. Descriptors			
		MAE	RMAE (%)	RMSE	SRCC	MAE	RMAE (%)	RMSE	SRCC
CH_4	log(k_H)	0.27	4.48	0.39	0.69	0.16	2.57	0.23	0.89
	Upt@5.8 bar	19.65	7.37	28.00	0.73	12.02	4.51	17.29	0.90
	Upt@65 bar	19.29	5.18	26.47	0.92	13.66	3.67	19.48	0.96
	Del. Cap.	14.02	4.88	19.23	0.91	10.84	3.78	15.40	0.94
CO_2	log(k_H)	0.72	8.04	1.00	0.49	0.42	4.66	0.62	0.83
	Upt@0.15 bar	0.96	10.29	1.38	0.51	0.54	5.80	0.80	0.84
	Upt@16 bar	0.89	2.62	1.35	0.96	0.58	1.73	0.95	0.98
Charges	MPC	0.27	9.59	0.39	0.45	0.06	2.16	0.13	0.95
	MNC	0.16	5.51	0.26	0.35	0.10	3.35	0.20	0.81

Supplementary Table 7. Accuracy of kernel ridge regression (KRR) models in prediction of gas adsorption properties of **BW-20K**. The machine learning models were trained using ~7,000 training data randomly chosen, and the statistics are reported for the remaining structures as the test set (~13,000 structures). All numbers were averaged over 10 different train-test splitting of the data. Units are similar to the main text. Henry coefficient (k_H), gas uptakes and deliverable capacity for CH_4, and gas uptakes for CO_2 are reported in mol.kg^{-1}.Pa^{-1}, vSTP/v, and mol.kg^{-1}, respectively. MAE: mean absolute error; RMAE: relative mean absolute error (%), and SRCC: Spearman ranking correlation coefficient.

	Property	Geo. Descriptors				Geo. & Chem. Descriptors			
		MAE	RMAE (%)	RMSE	SRCC	MAE	RMAE (%)	RMSE	SRCC
CH_4	log(k_H)	0.17	4.24	0.25	0.79	0.14	3.35	0.21	0.87
	Upt@5.8 bar	11.54	6.21	16.36	0.90	8.80	4.74	11.96	0.94
	Upt@65 bar	14.35	4.90	18.68	0.93	10.88	3.72	14.64	0.96
	Del. Cap.	9.90	4.39	13.12	0.97	9.90	4.39	13.12	0.97
CO_2	log(k_H)	0.31	4.60	0.45	0.82	0.24	3.57	0.36	0.89
	Upt@0.15 bar	0.43	5.21	0.64	0.83	0.30	3.59	0.45	0.92
	Upt@16 bar	1.15	3.33	1.78	0.98	0.74	2.16	1.11	0.99
Charges	MPC	0.11	4.92	0.16	0.63	0.05	2.01	0.07	0.90
	MNC	0.10	3.88	0.12	0.35	0.07	2.70	0.09	0.71

Supplementary Table 8. Accuracy of random forest regression (RF) models in prediction of gas adsorption properties of **BW-20K**. The machine learning models were trained using ~7,000 training data randomly chosen, and the statistics are reported for the remaining structures as the test set (~13,000 structures). All numbers were averaged over 10 different train-test splitting of the data. Units are similar to the main text. Henry coefficient (k_H), gas uptakes and deliverable capacity for CH_4, and gas uptakes for CO_2 are reported in mol.kg^{-1}.Pa^{-1}, vSTP/v, and mol.kg^{-1}, respectively. MAE: mean absolute error; RMAE: relative mean absolute error (%), and SRCC: Spearman ranking correlation coefficient.

	Property	Geo. Descriptors				Geo. & Chem. Descriptors			
		MAE	RMAE (%)	RMSE	SRCC	MAE	RMAE (%)	RMSE	SRCC
CH_4	log(k_H)	0.17	4.12	0.24	0.79	0.12	3.02	0.19	0.88
	Upt@5.8 bar	11.58	6.23	16.46	0.89	8.69	4.68	12.53	0.93
	Upt@65 bar	14.27	4.87	18.67	0.93	11.57	3.95	15.43	0.95
	Del. Cap.	10.03	4.44	13.22	0.97	8.31	3.68	11.16	0.98
CO_2	log(k_H)	0.31	4.59	0.45	0.81	0.23	3.31	0.33	0.90
	Upt@0.15 bar	0.44	5.29	0.66	0.83	0.32	3.80	0.48	0.91
	Upt@16 bar	1.16	3.36	1.78	0.98	0.86	2.50	1.29	0.99
Charges	MPC	0.11	4.75	0.16	0.64	0.03	1.43	0.05	0.94
	MNC	0.10	3.90	0.12	0.34	0.07	2.80	0.10	0.68

Supplementary Table 9. Accuracy of gradient boosting regression (GBR) models in prediction of gas adsorption properties of **BW-20K**. The machine learning models were trained using ~7,000 training data randomly chosen, and the statistics are reported for the remaining structures as the test set (~13,000 structures). All numbers were averaged over 10 different train-test splitting of the data. Units are similar to the main text. Henry coefficient (k_H), gas uptakes and deliverable capacity for CH_4, and gas uptakes for CO_2 are reported in mol.kg^{-1}.Pa^{-1}, vSTP/v, and mol.kg^{-1}, respectively. MAE: mean absolute error; RMAE: relative mean absolute error (%), and SRCC: Spearman ranking correlation coefficient.

	Property	Geo. Descriptors				Geo. & Chem. Descriptors			
		MAE	RMAE (%)	RMSE	SRCC	MAE	RMAE (%)	RMSE	SRCC
CH_4	log(k_H)	0.17	4.22	0.25	0.79	0.12	2.97	0.18	0.89
	Upt@5.8 bar	11.80	6.35	16.66	0.89	8.08	4.35	11.42	0.94
	Upt@65 bar	14.34	4.90	18.65	0.93	10.73	3.66	14.31	0.96
	Del. Cap.	10.11	4.48	13.30	0.97	8.08	3.58	10.78	0.98
CO_2	log(k_H)	0.32	4.63	0.46	0.81	0.23	3.31	0.34	0.90
	Upt@0.15 bar	0.44	5.31	0.68	0.82	0.32	3.85	0.50	0.91
	Upt@16 bar	1.17	3.40	1.80	0.98	0.76	2.21	1.12	0.99
Charges	MPC	0.11	4.73	0.17	0.65	0.03	1.43	0.05	0.95
	MNC	0.10	3.92	0.13	0.31	0.07	2.71	0.10	0.68

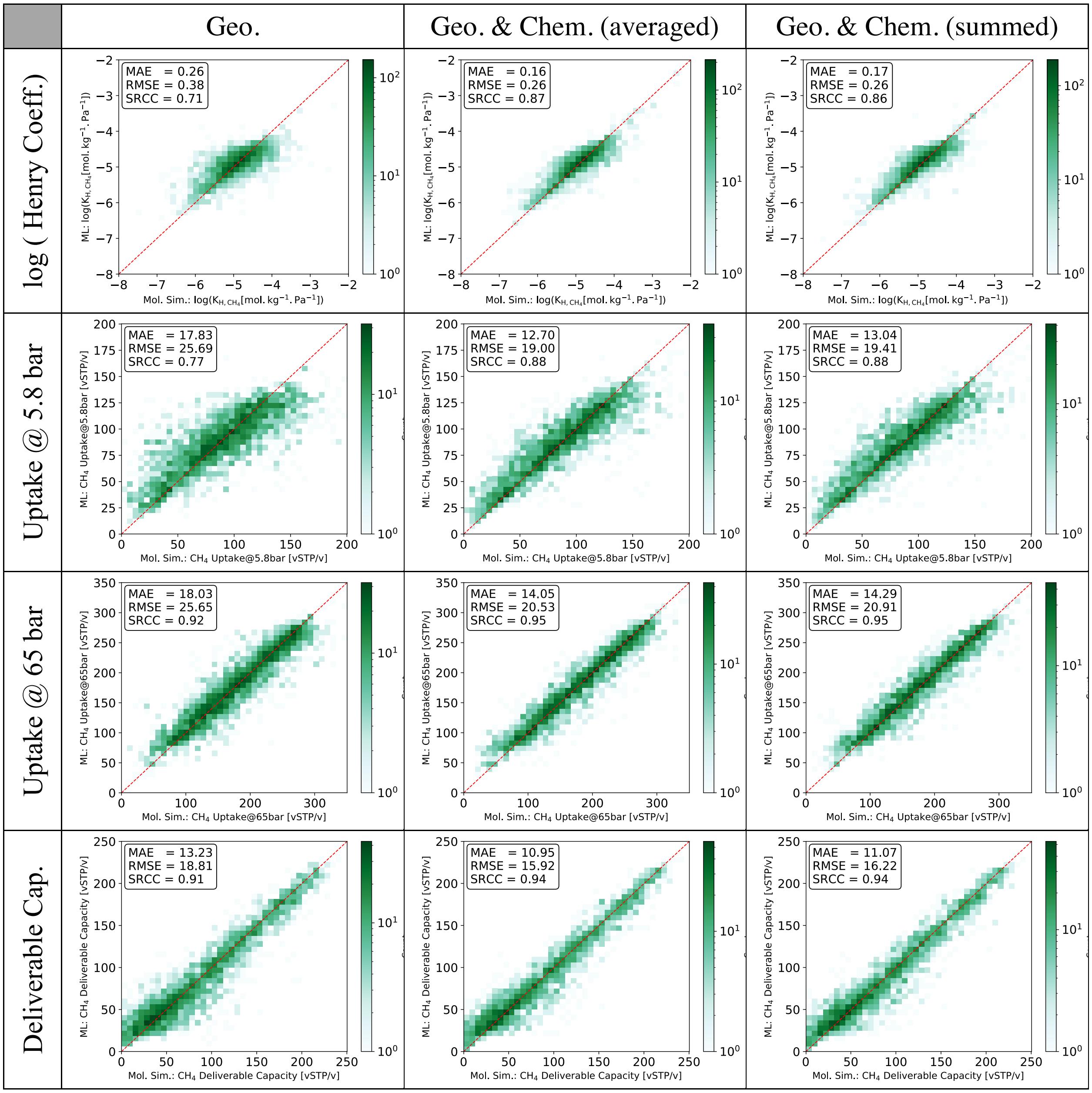

Supplementary Figure 1. Two-dimensional histogram parity plots and statistics of the accuracy of random forest regression in predictions of the CH_4 adsorption properties for the test set from **CoRE-2019**. ~7,000 structures were used for training and the remaining ~2,500 structures were used for test. Statistics were reported as average over 10 separate random seeds for train-test splitting. Color-bar shows number of structures in each cell of the histogram.

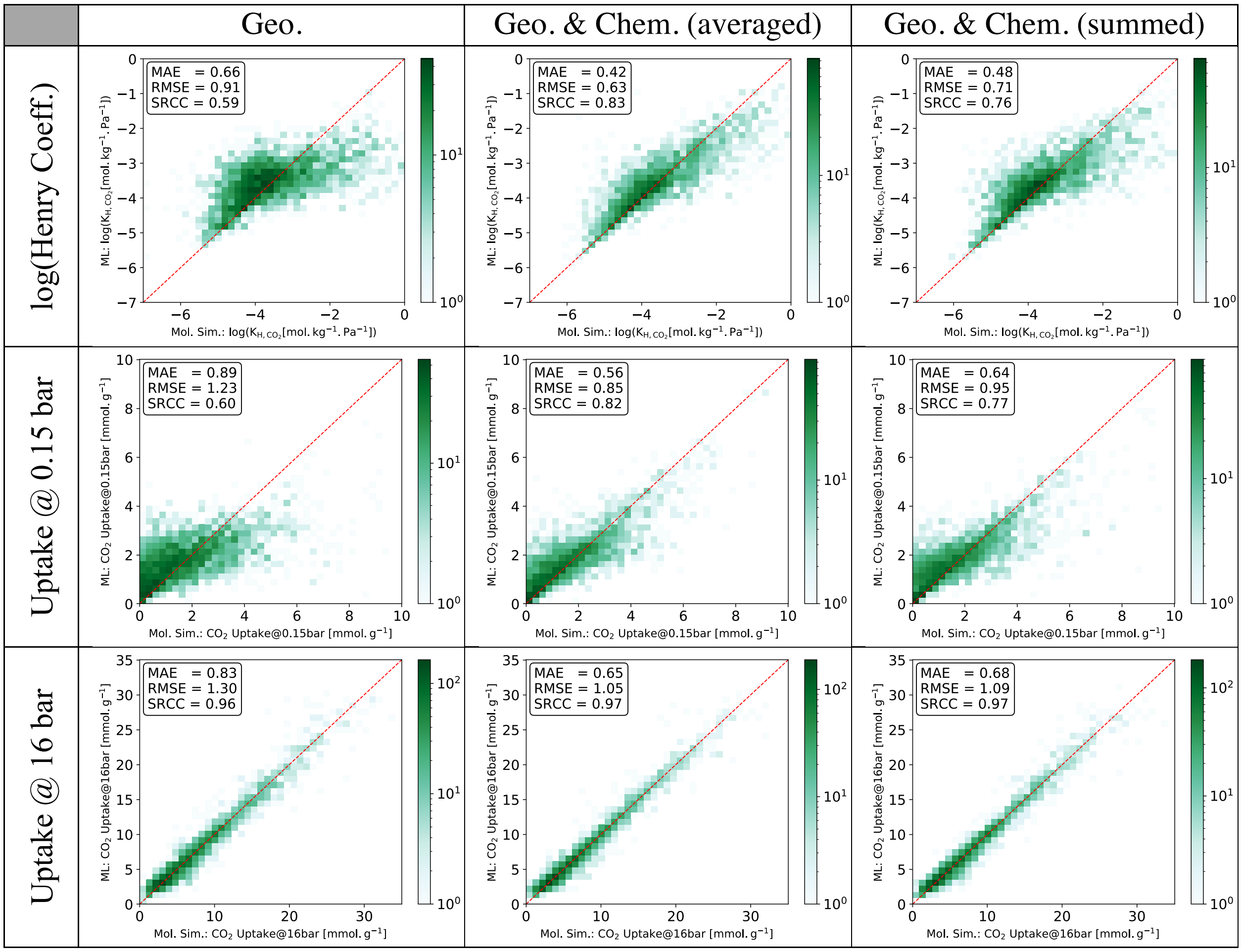

Supplementary Figure 2. Two-dimensional histogram parity plots and statistics of the accuracy of random forest regression in predictions of the CO_2 adsorption properties for the test set from **CoRE-2019**. ~7,000 structures were used for training and the remaining ~2,500 structures were used for test. Statistics were reported as average over 10 separate random seeds for train-test splitting. Color-bar shows number of structures in each cell of the histogram.

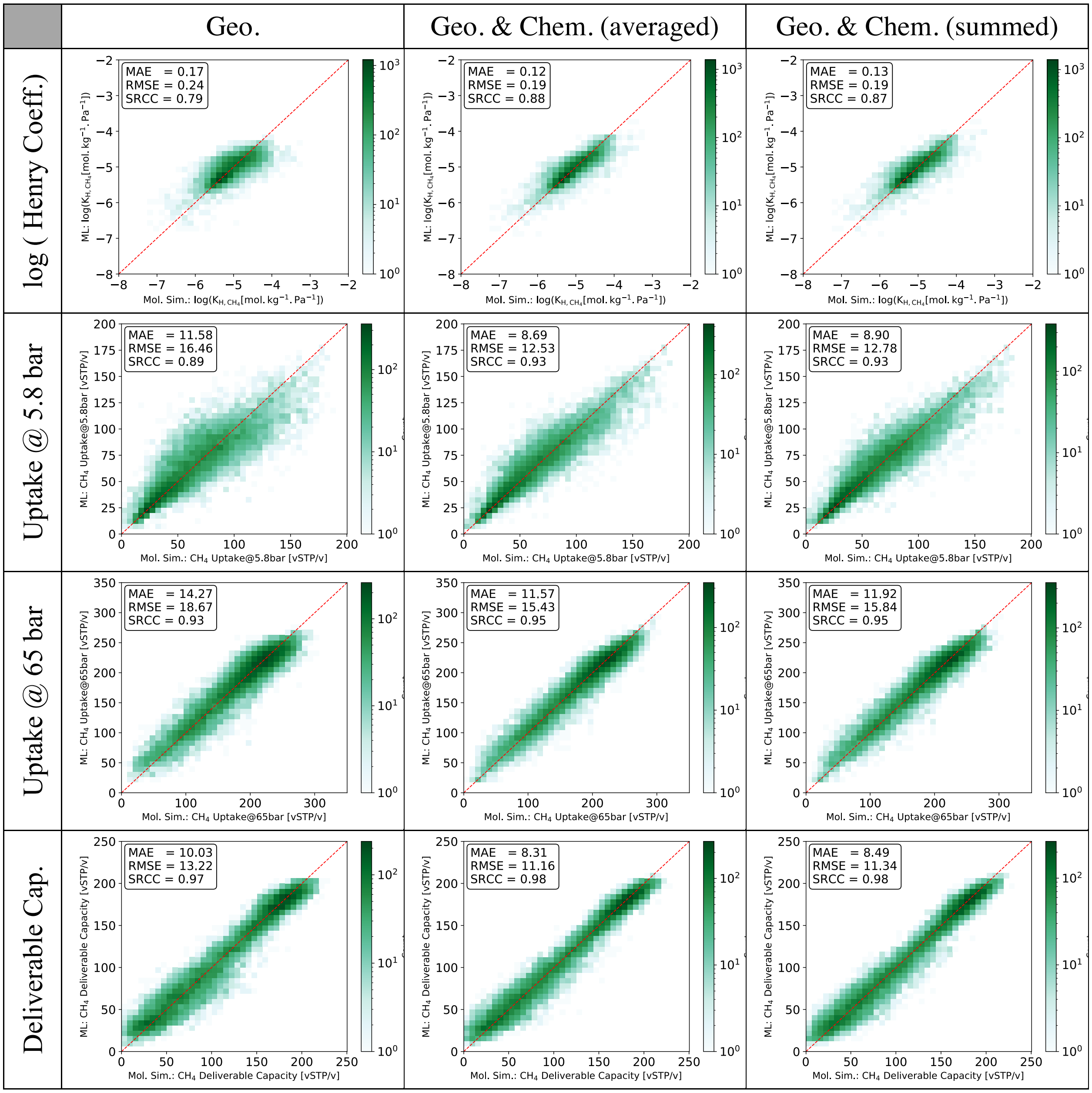

Supplementary Figure 3. Two-dimensional histogram parity plots and statistics of the accuracy of random forest regression in predictions of the CH_4 adsorption properties for the test set from **BW-20K**. ~7,000 structures were used for training and the remaining ~13,000 structures were used for test. Statistics were reported as average over 10 separate random seeds for train-test splitting. Color-bar shows number of structures in each cell of the histogram.

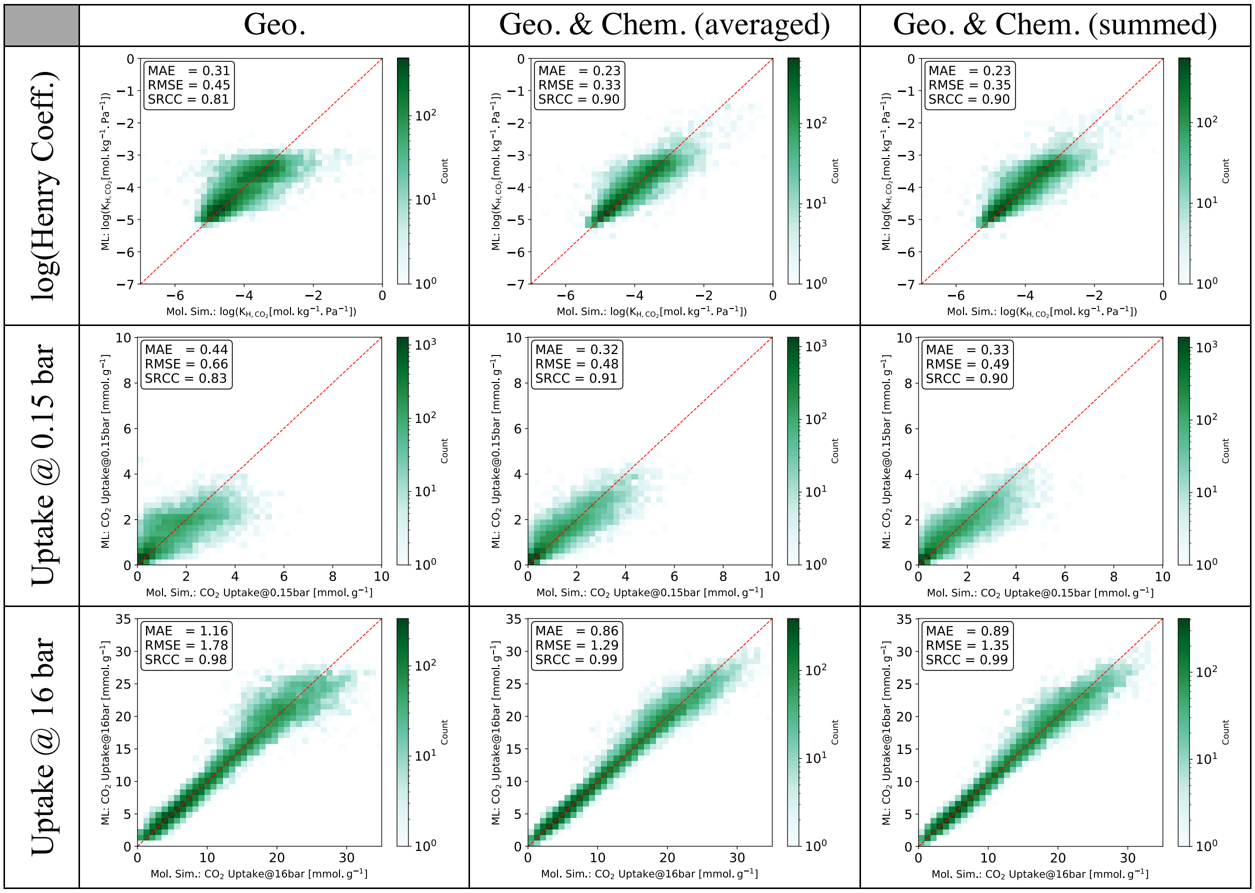

Supplementary Figure 4. Two-dimensional histogram parity plots and statistics of the accuracy of random forest regression in predictions of the CO_2 adsorption properties for the test set from **BW-20K**. ~7,000 structures were used for training and the remaining ~13,000 structures were used for test. Statistics were reported as average over 10 separate random seeds for train-test splitting. Color-bar shows number of structures in each cell of the histogram.

Supplementary Note 4: Partial Atomic Charges

The partial atomic charges for the CoRE MOF database (**CoRE-2019**)[4,5] were derived using the extended charge equilibration (EQeq) method.[11,12] We use random forest regression models to predict the maximum positive charge (MPC) and minimum negative charge (MNC) of the frameworks using only geometric, and geometric and chemical descriptors. We observe the chemical descriptors are able to learn and predict these attributes of the MOF structures in the **CoRE-2019** with high accuracies.

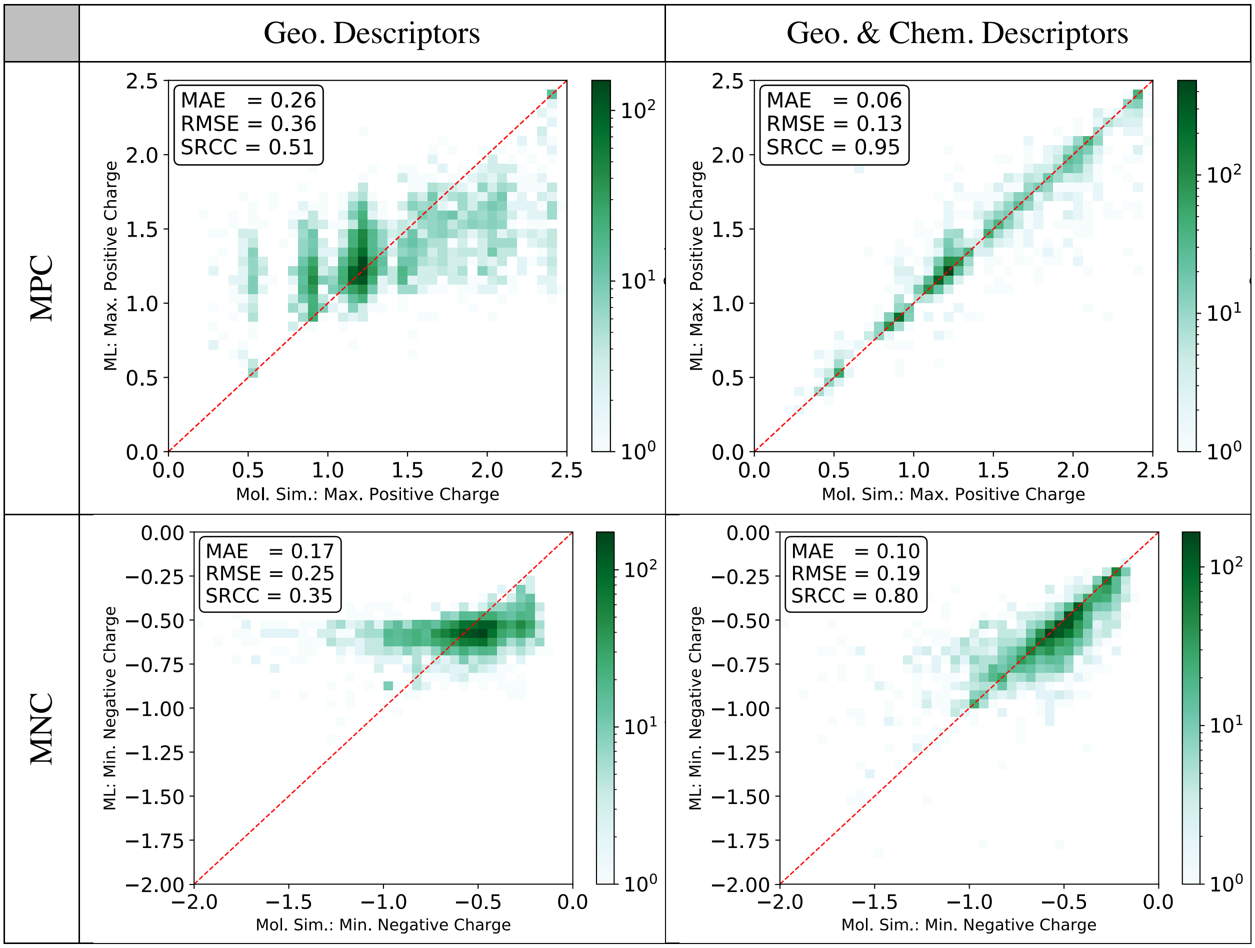

Supplementary Figure 5. Two-dimensional histogram parity plots and statistics of the accuracy in machine learning predictions of the framework maximum positive charge (MPC) and minimum negative charge (MNC) using geometric or geometric and chemical descriptors for test set from **CoRE-2019**. Partial atomic charges were derived using EQeq method for this database. Random forest regressions were trained using ~7,000 structures and the remaining structures (~2,500 structures) were used as test set. Statistics were reported as average over 10 separate random seeds for train-test splitting. Color-bar shows number of structures in each cell of the histogram.

For a subset of the structures in the CoRE MOF database (~2900 structures – **CoRE-DDEC**), Nazarian et al.[6] performed DFT calculations and derived DDEC charges. Comparing DDEC charges with EQeq is instructive. The correlation between these charges are poor which shows the intrinsic problem with machine learning representations using method dependent features (see Supplementary Figure 6). We see in Supplementary Figure 7 that our chemical descriptors are able to learn the charges derived with both EQeq and DDEC approaches. We note that our prediction accuracies are higher for DDEC charges. This might be due to more smooth behaving of the DFT derived charges which ease the learning process.

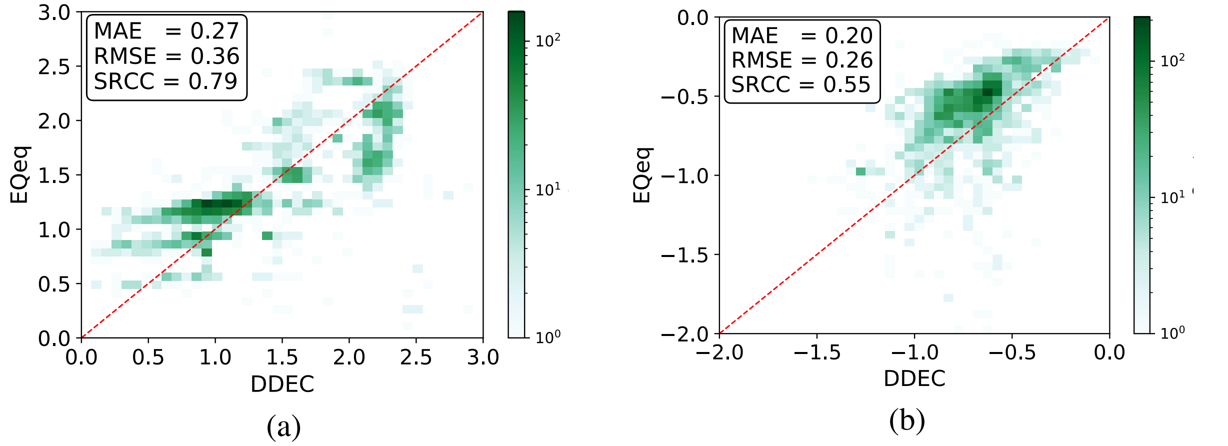

Supplementary Figure 6. Two-dimensional histogram parity plots and statistics of correlations between two methods for deriving partial atomic charges, namely extended charge equilibration (EQeq) and density derived electrostatics and chemical (DDEC) methods. (a) maximum positive charge of the framework and (b) minimum negative charge of the framework.

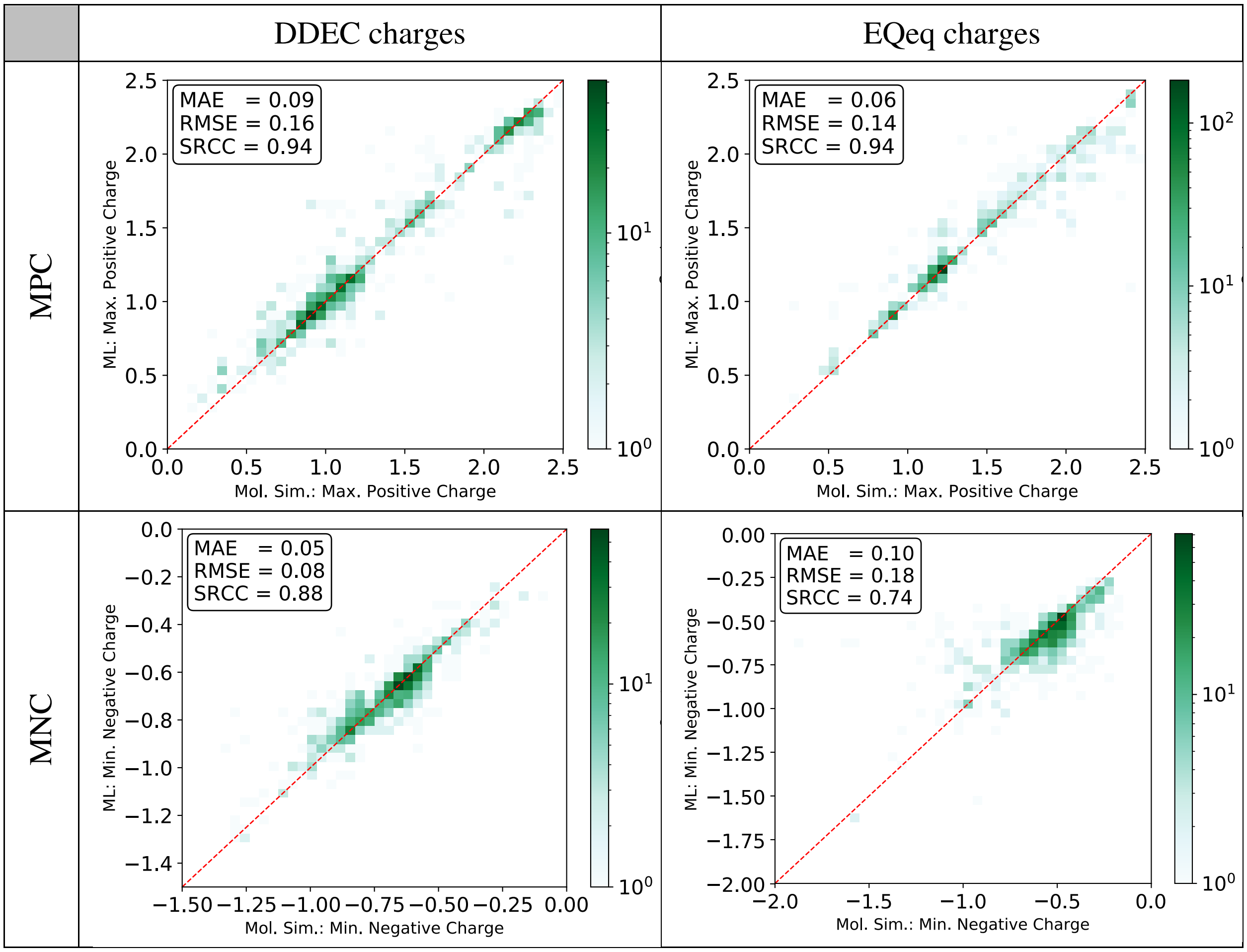

Supplementary Figure 7. Two-dimensional histogram parity plots and statistics of the accuracy in machine learning predictions of the framework maximum positive charge (MPC) and minimum negative charge (MNC) derived from two different approaches, DDEC and EQeq, for the **CoRE-DDEC**. The DDEC charges were obtained from ref.[6] Random forest regressions were trained using ~2,000 structures and the remaining structures (~800 structures) were used as test set. Statistics were reported as average over 10 separate random seeds for train-test splitting. Color-bar shows number of structures in each cell of the histogram.

Supplementary Note 5: Importance of Variables for Gas Adsorption Properties

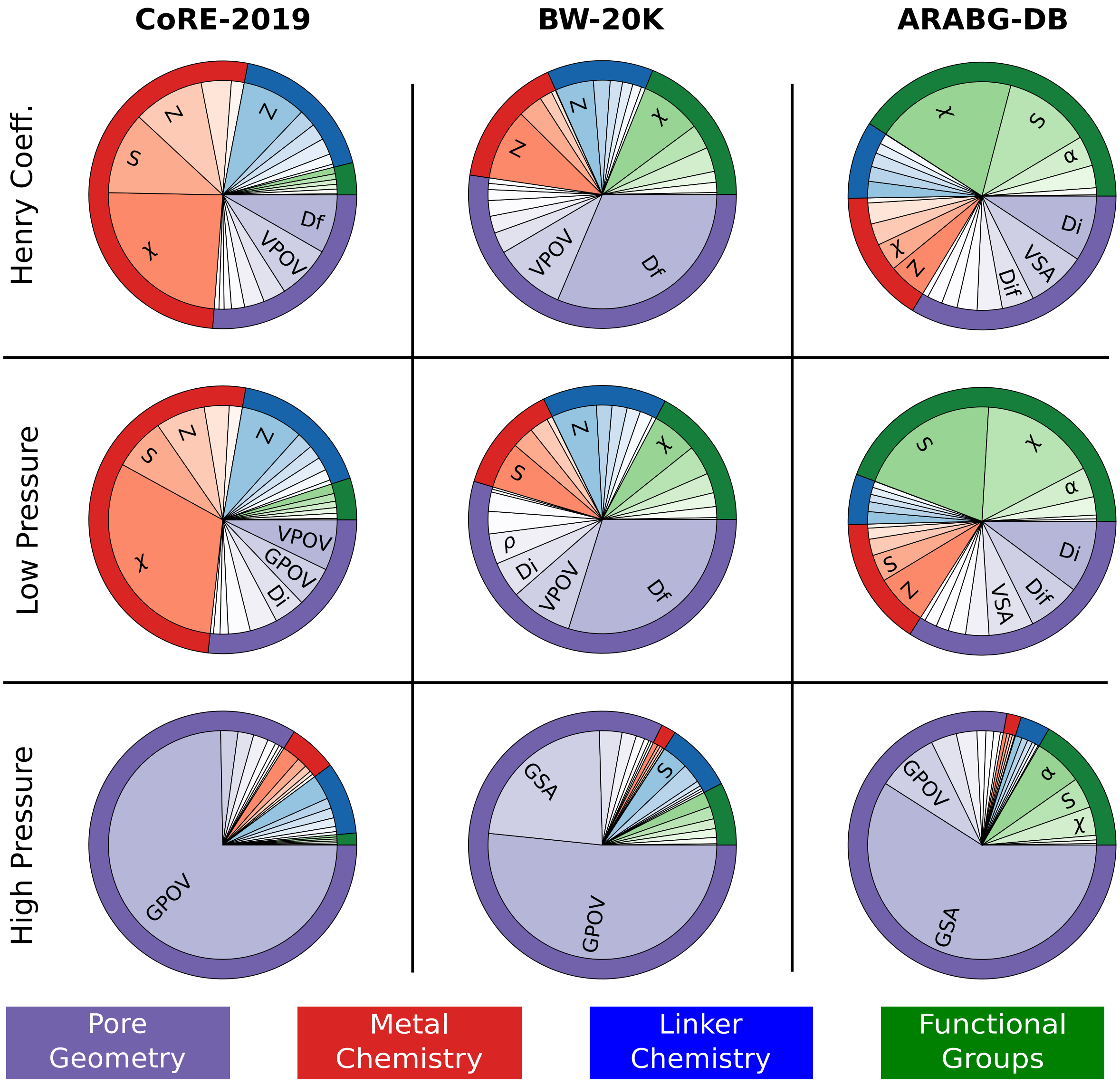

Supplementary Figure 8. **Feature importance for CO_2 adsorption properties.** Pie charts showing the SHAP values (importance of variables). SHAP values were computed for the random forest regression models using a training set of **CORE-2019** and **BW-20K**, and all structures in **ARABG-DB**. For the chemical features, the importance of variables was summed over all RAC depths for each of the heuristic atomic properties. See method section for the meaning of the labels.

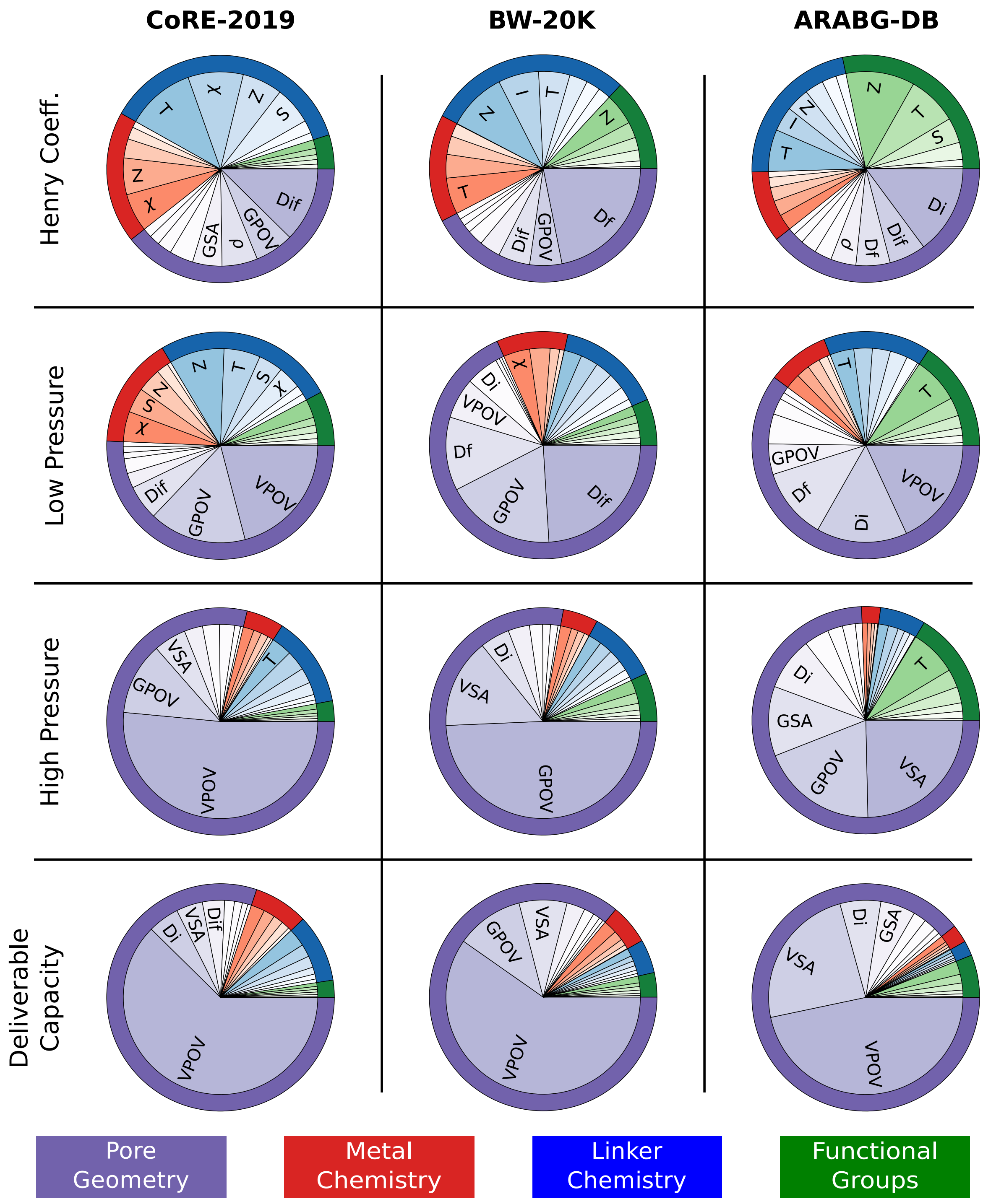

Supplementary Figure 9. **Feature importance for CH_4 adsorption properties.** Pie charts showing the SHAP values (importance of variables). SHAP values were computed for the random forest regression models using a training set of **CORE-2019** and **BW-20K**, and all structures in **ARABG-DB**. For the chemical features, the importance of variables was summed over all RAC depths for each of the heuristic atomic properties. See method section for the meaning of the labels.

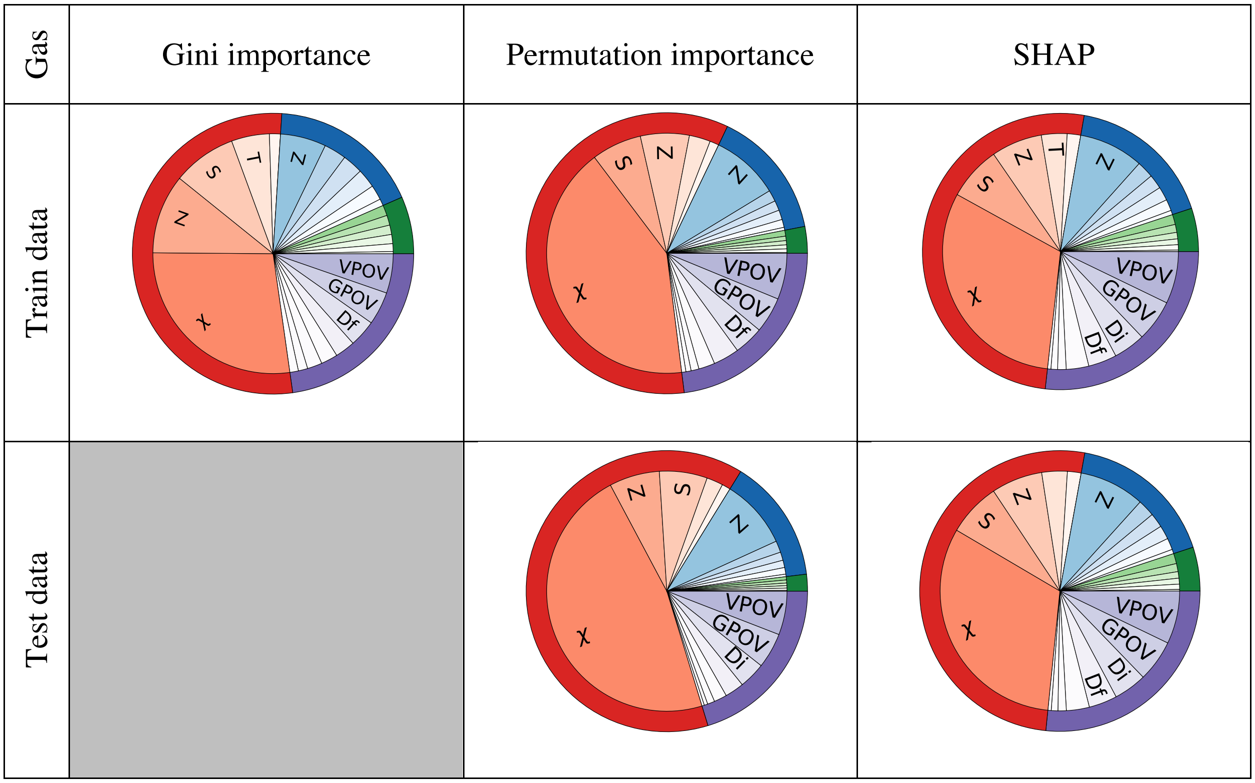

Supplementary Figure 10. Comparing different methods for evaluating importance of variables for the low-pressure CO_2 adsorption in materials in **CoRE-2019**. The pie charts are color-coded with MOF material domains; purple: pore geometry, red: metal chemistry, blue: linker chemistry, and green: functional groups. For the chemical features, the importance of variables was summed over all RAC depths for each heuristic atomic property.

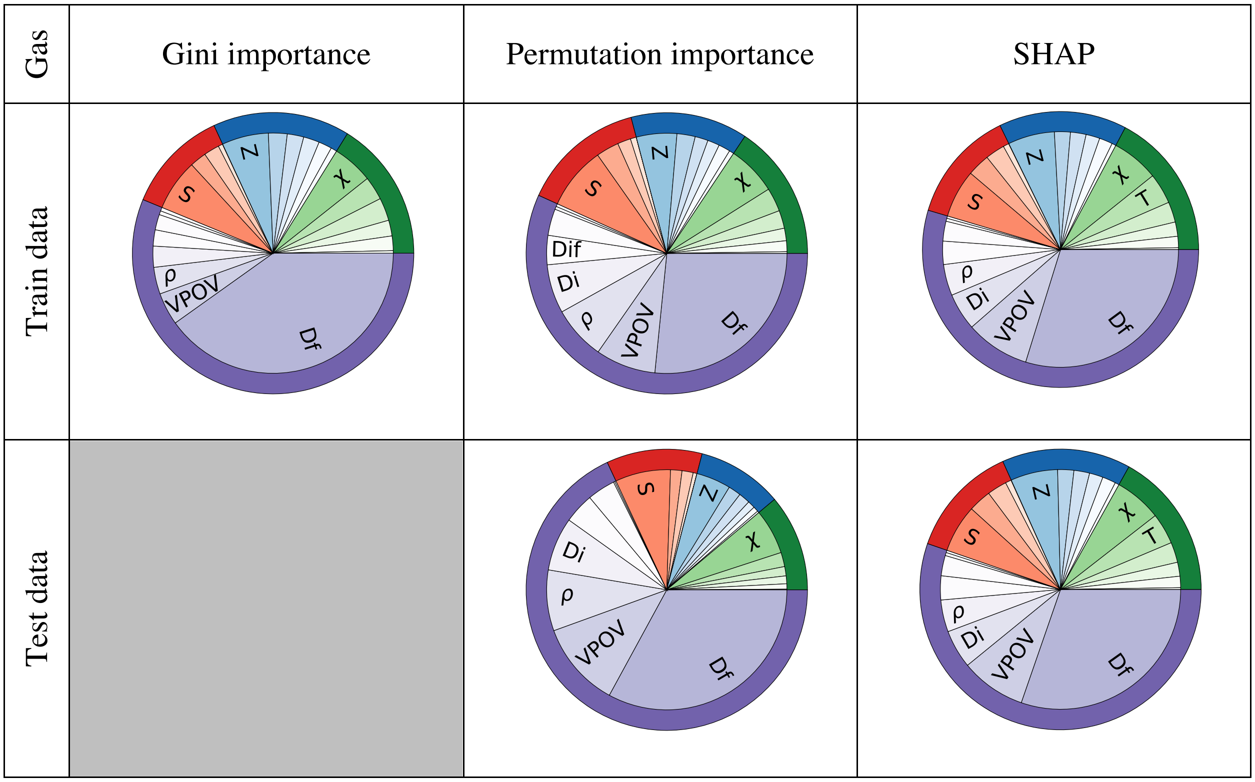

Supplementary Figure 11. Comparing different methods for evaluating importance of variables for the low-pressure CO_2 adsorption in materials in **BW-20K**. The pie charts are color-coded with MOF material domains; purple: pore geometry, red: metal chemistry, blue: linker chemistry, and green: functional groups. For the chemical features, the importance of variables was summed over all RAC depths for each heuristic atomic property.

<table>
<tr><th>Gini importance</th><th>Permutation importance</th><th>SHAP</th></tr>
<tr>
<td>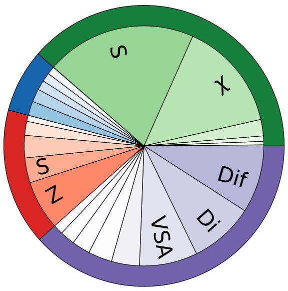</td>
<td>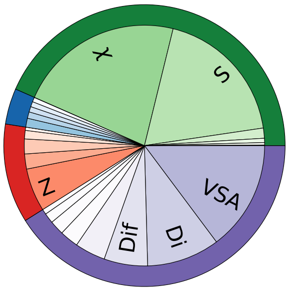</td>
<td>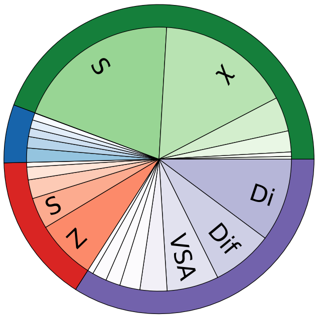</td>
</tr>
</table>

Supplementary Figure 12. Comparing different methods for evaluating importance of variables for the low-pressure CO_2 adsorption in materials in **ARABG-DB** using all the materials in the database. The pie charts are color-coded with MOF material domains; purple: pore geometry, red: metal chemistry, blue: linker chemistry, and green: functional groups. For the chemical features, the importance of variables was summed over all RAC depths for each heuristic atomic property.

Supplementary Note 6: PCA/TSNEs

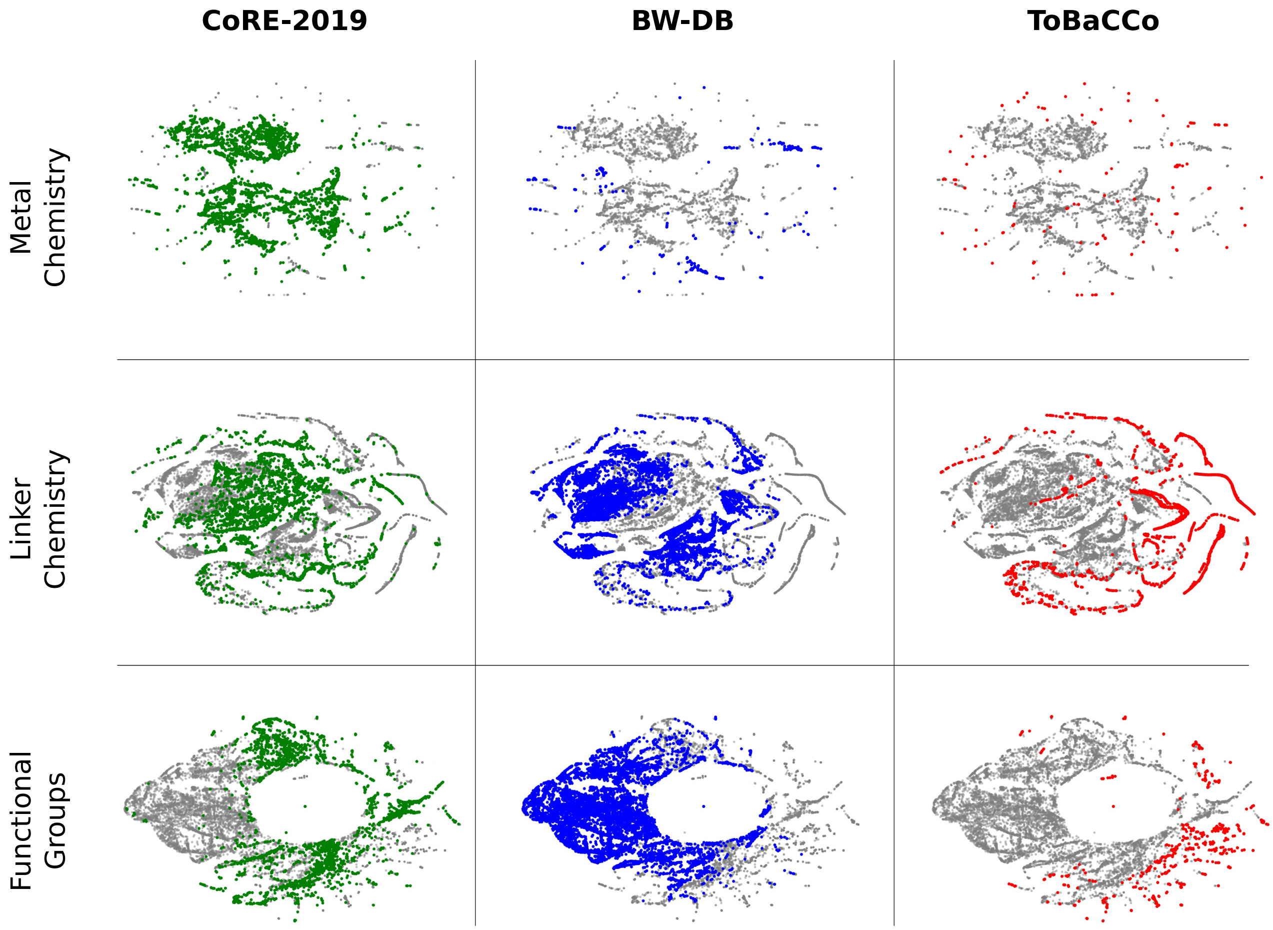

Supplementary Figure 13. The t-SNE maps showing the distribution of the materials in each database. Each database is overlaid using colored dots over the current chemical space that is shown in gray.

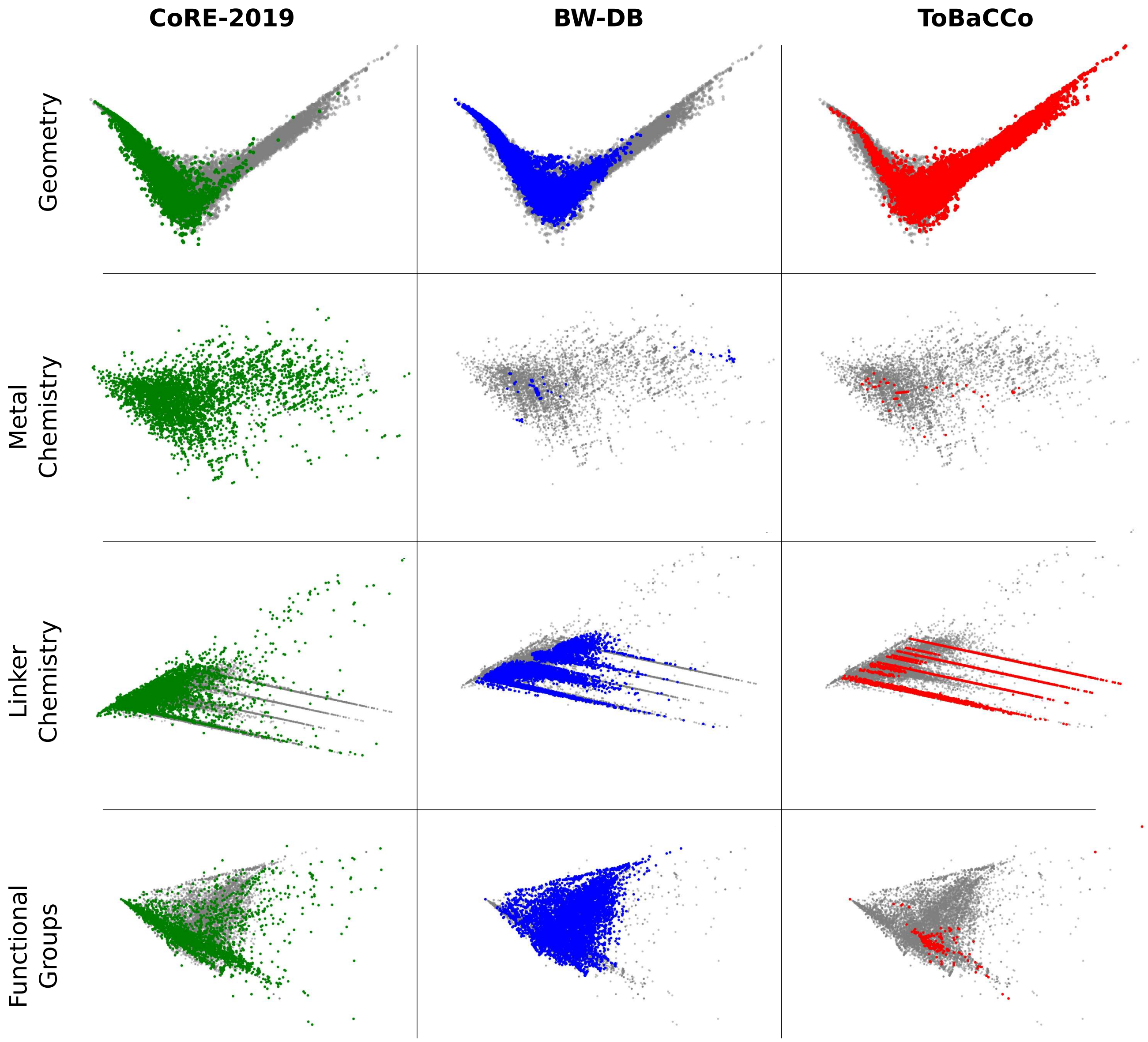

Supplementary Figure 14. The PCA maps showing the distribution of the materials in each database. Each database is overlaid using colored dots over the current chemical space that is shown in gray.

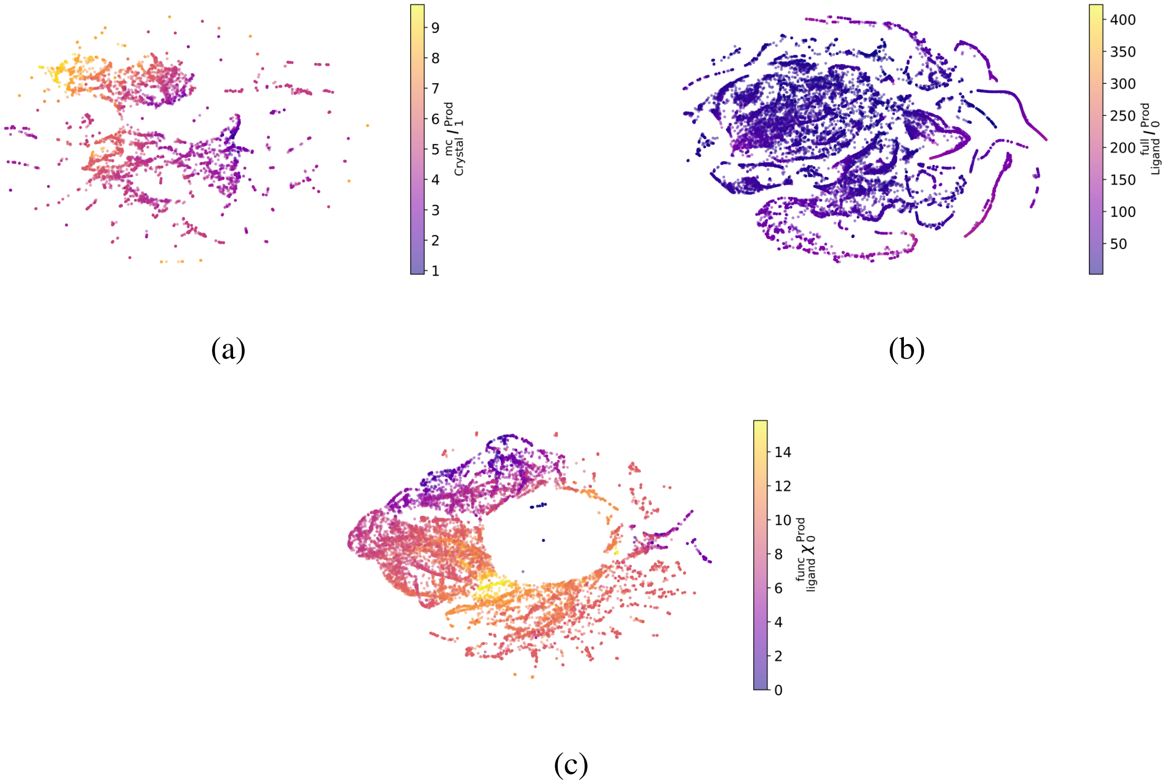

Supplementary Figure 15. The t-SNE representation of different domains of MOF chemistry. (a) metal center descriptors color-coded with the coordination number of the metal (b) linker chemistry color-coded with size of the linker, and (c) functional groups color-coded with electronegativity of the group.

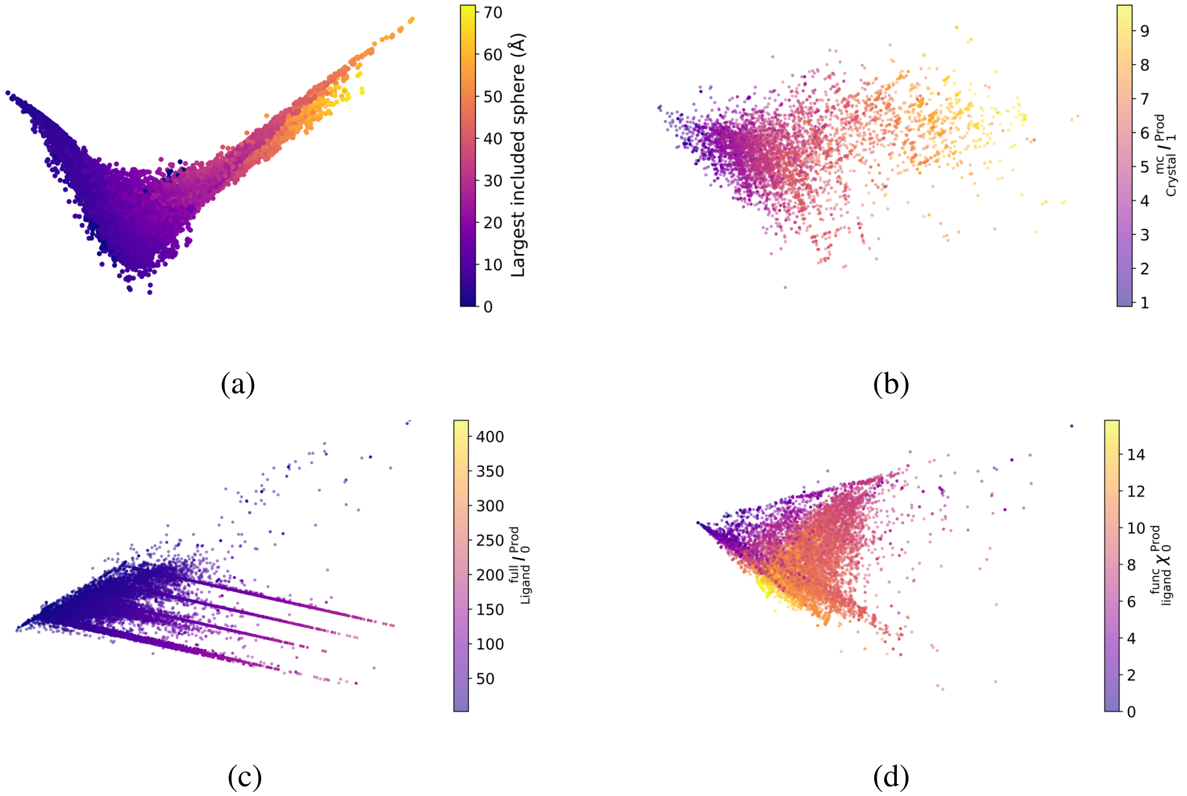

Supplementary Figure 16. The PCA representation of the different domains of MOF descriptors. (a) geometric descriptors color-coded with size of the pore, (b) metal center descriptors color-coded with the coordination number of the metal (c) linker chemistry color-coded with size of the linker, and (d) functional groups color-coded with electronegativity of the group.

Supplementary Note 7: Mining Metal Nodes from CoRE-MOF Database

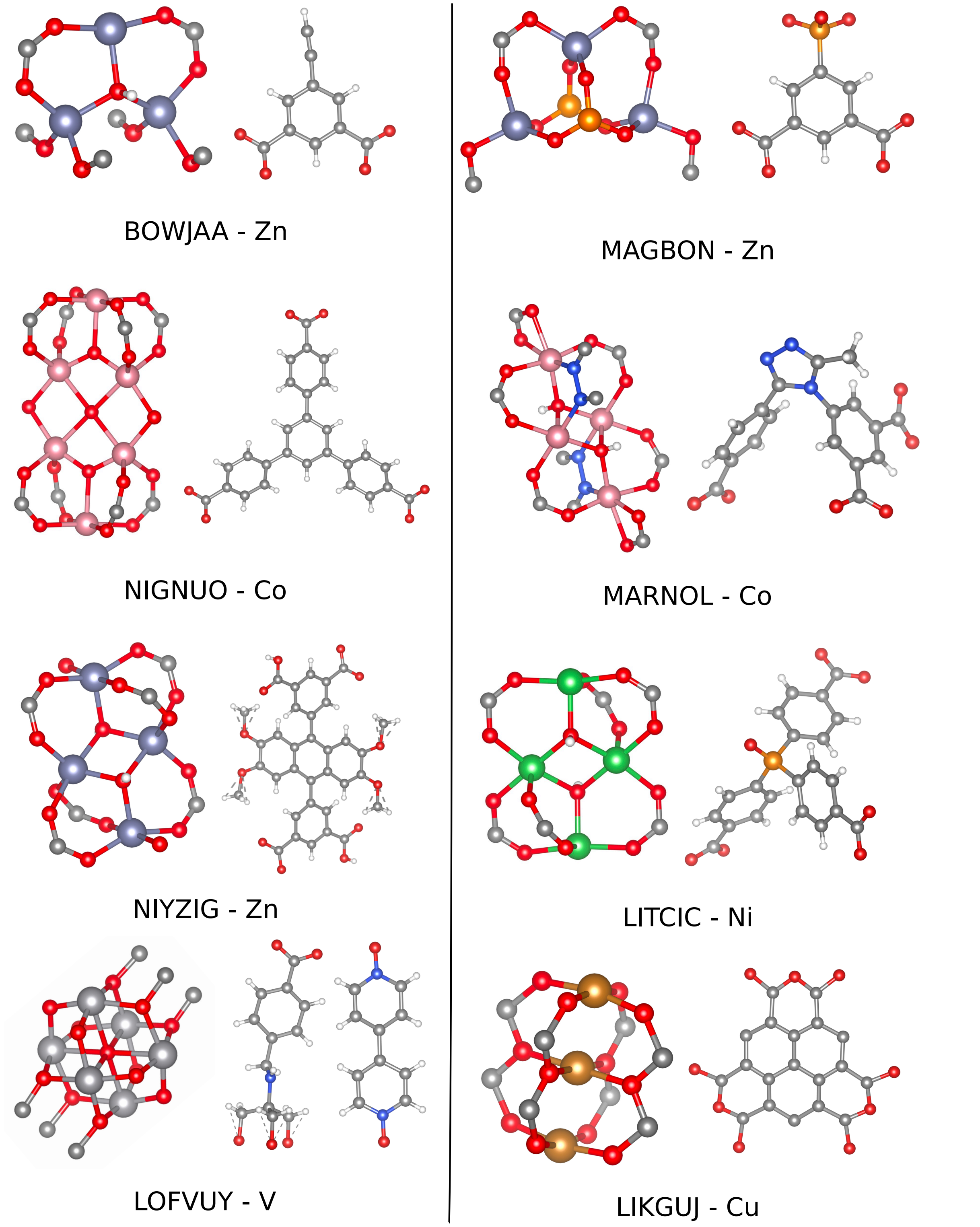

Supplementary Figure 17. Inorganic SBUs mined from CoRE-2019 and their corresponding linkers. These are examples of inorganic SBUs that are missing in hypothetical MOF databases. The CSD names and metal types are shown below each

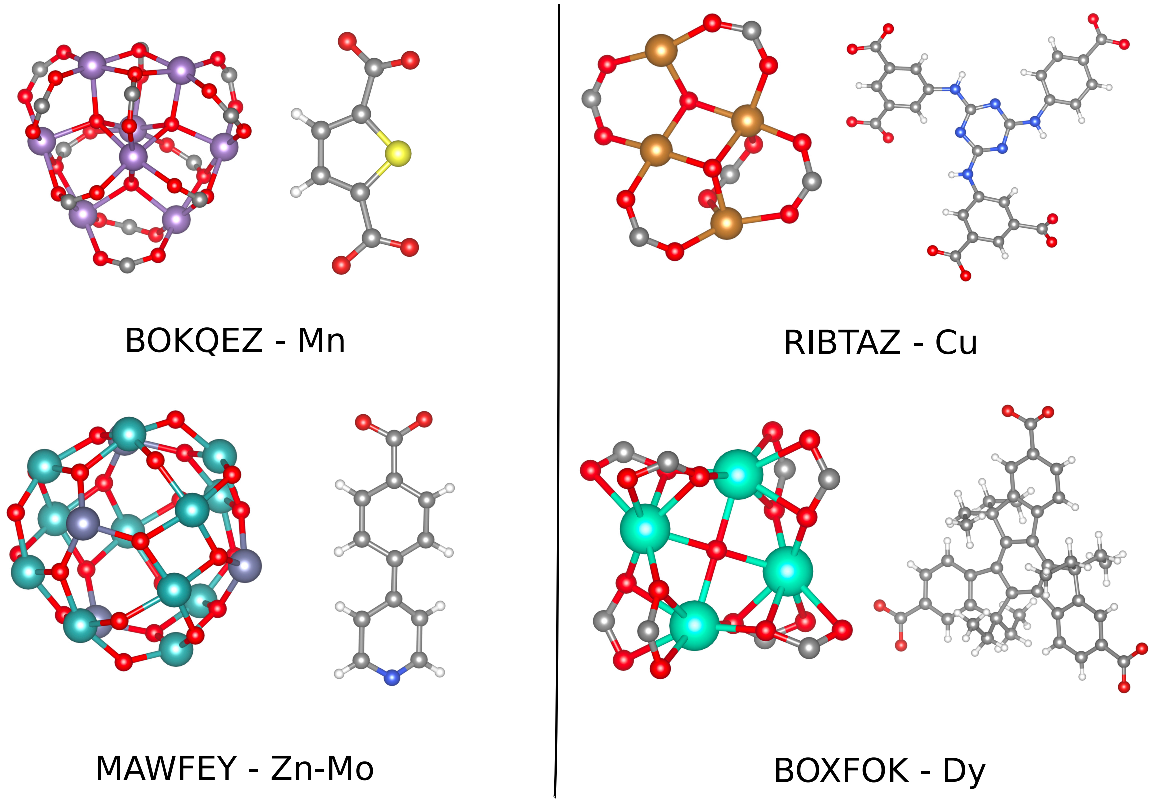

Supplementary Figure 18. Inorganic SBUs mined from CoRE-2019 and their corresponding linkers. These are examples of inorganic SBUs that are missing in hypothetical MOF databases. The CSD names and metal types are shown below each SBU.

Supplementary Note 8: Notes on KRR Models

The hyperparameters of KRR models were selected based on recursive feature addition (RFA) or threshold explained variance methods. For the RFA method,[13,14] we use the ranking based on Gini importance[15] of variables derived from random forest models. We keep adding features until the improvement in CV score remains below 0.1% (see figure below as an example). For the explained variance method, we use threshold of 0.95 for feature selection. For hyperparameter optimization we use a combination of Tree of Parzen Estimators (TPE), annealing, and random search with a ratio of 0.7,0.15, and 0.15, respectively, with maximum 150 evaluation. The hyperparameters for the models and the number of selected features is show in Tables below.

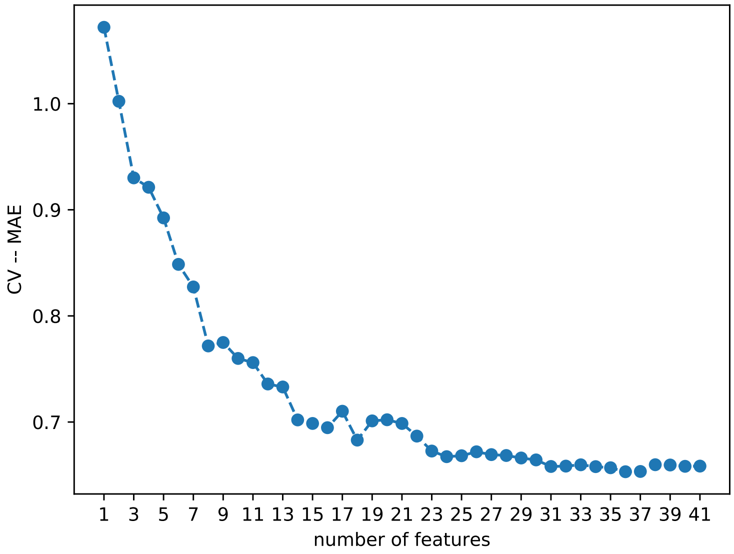

Supplementary Figure 19. The cross-validation score optimization using RFA method.

Supplementary Table 10. The hyperparameters and number of selected features for training KRR models to predict different properties of **BW20K**. kH: Henry coefficient, LP: low pressure, HP: high pressure, DC: deliverable capacity, MPC: maximum positive charge, MNC: minimum negative charge.

	CH_4				CO_2			Charge	
	kH	LP	HP	DC	kH	LP	HP	MPC	MNC
α	0.0025	0.0021	0.0070	0.0039	0.0232	0.0846	0.0149	0.0034	0.0021
γ	0.0007	0.0014	0.0024	0.2029	0.0025	0.0388	0.0096	0.0001	0.0047
N features	35	36	41	7	28	33	36	41	40

Supplementary Table 11. The hyperparameters and number of selected features for training KRR models to predict different properties of **CoRE2019**. kH: Henry coefficient, LP: low pressure, HP: high pressure, DC: deliverable capacity, MPC: maximum positive charge, MNC: minimum negative charge.

	CH_4				CO_2			Charge	
	kH	LP	HP	DC	kH	LP	HP	MPC	MNC
α	0.0030	0.0419	0.0103	0.0089	0.0030	0.1586	0.0068	0.0866	0.2822
γ	0.0088	0.0286	0.0127	0.0649	0.0086	0.0820	0.0898	2.0313	0.0634
N features	39	41	33	11	39	36	5	4	26

Supplementary Note 9: Notes on RF and GBR models

For the gradient boosted regressor (GBR) and random forest (RF) models we use all the features. To find the hyperparameters of the models, we perform exhaustive grid search[14] over the grids of parameters shown in the lists below:

a) GBR:
   1. Learning rate: [0.001,0.01,0.05,0.1,0.2,0.5,1.0]
   2. Number of trees: [50,100,200,300]
   3. Subsample: [0.9,1.0]
   4. Minimum split: [2,3]
   5. Max depth: [3,4,5]

b) RF:
   1. Max depth: [5,10,20,40]
   2. Number of trees: [50,100,150,200,250,300]

Supplementary Note 10: Structure Refining Steps

To prepare the data for featurization and gas adsorption calculations, we carried out a series of steps for cleaning the databases we studied (see figure below). As a first step, we check if the occupancies of the .cif file is correct while parsing the structures. We exclude all the .cif files that are too large, or they do not contain any metals. Then, we compute the periodic pairwise distance matrix between all atoms of the framework and identified cases with atomic overlap when the pairwise distance between two atoms is less than the covalent radii of each atom. After assigning the adjacency matrix (See *Supplementary Note 1*), we check each of the connected components of this matrix, and the structures with a connected component that does not contain a metal are identified with having floating atoms (e.g., a solvent molecule) and excluded. If a structure passes all these steps, we perform geometric and RACs featurization for it.

The next step is to filter materials for gas adsorption calculation. All the structures that are non-porous to a probe radius of 1.86 Å are excluded for the gas adsorption calculations. We perform partial atomic charges assignment in this step. The structures that take framework maximum positive charge bigger than 3 or minimum positive charge smaller than -3 are recognized to be unrealistic and were excluded. The number of structures from each of the databases studied in this work that passed this database refinement protocol are listed in Supplementary Table 12.

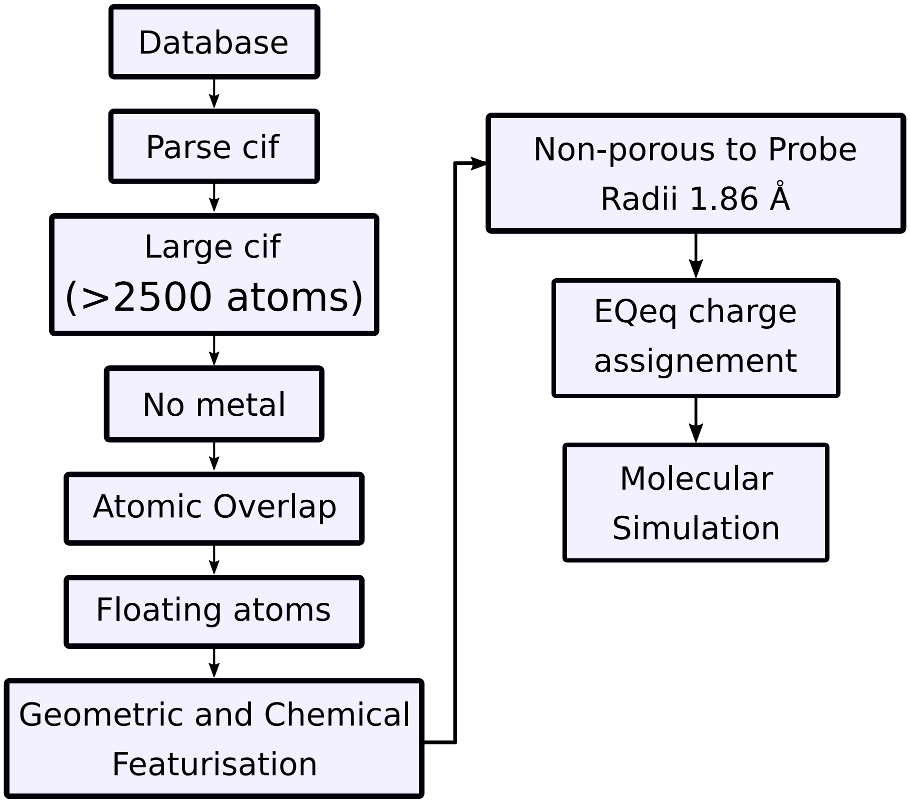

Supplementary Figure 20 A flowchart representation of the database refinement carried out in this study.

Supplementary Table 12. The number of structures from each database that pass the refinement steps and the final dataset sizes.

Database	Number of Structures	Cif Parse Failure	Large cif	No Metal	Atomic Overlap	Floating Atoms	RACs Failed	Geo. Feat. Failed	Featurized	nonporous	EQeq failed	Mol. Sim. Failed	All data
CoRE-2019	11920	0	60	4	1108	136	4	2	10606	752	163	166	9525
CoRE-DDEC	2932	1	0	3	42	78	0	0	2808	313	13	0	2795
hMOF	137953	0	0	0	1786	15527	0	45	120595	8418			
BW-DB	324426	133	5	0	159	367	0	136	323626	4032			
ToBaCCo	13514	4	1354	301	133	179	0	0	11543	13			
BW-20K	20000	77	0	0	20	30	0	0	19873	484	4	6	19387
ARABG-DB	426	0	37	0	0	0	0	0	389	2	0	0	387

Supplementary Note 11: Molecular Simulation Parameter Sets

Supplementary Table 13. The Lennard-Jones parameters of the framework atoms extracted from UFF[16] and TraPPE[17] force fields.

Atom	ε [K]	σ(Å)	Atom	ε [K]	σ(Å)	Atom	ε [K]	σ(Å)	Atom	ε [K]	σ(Å)
**Ac**	16.608	3.0985	**Cm**	6.5425	2.9631	**Ho**	3.5229	3.0371	**Sc**	9.5622	2.9355
**Ag**	18.1178	2.8045	**Co**	7.0458	2.5587	**I**	170.609	4.009	**Se**	146.4519	3.7462
**Al**	254.152	4.0082	**Cr**	7.5491	2.6932	**In**	301.4595	3.9761	**Si**	202.315	3.8264
**Am**	7.0458	3.0121	**Cs**	22.6472	4.0242	**Ir**	36.7388	2.5302	**Sm**	4.0262	3.136
**Ar**	93.1052	3.446	**Cu**	2.5164	3.1137	**K**	17.6145	3.3961	**Sn**	285.3548	3.9128
**As**	155.5108	3.7685	**Dy**	3.5229	3.054	**Kr**	110.7197	3.6892	**Sr**	118.2687	3.2438
**At**	142.929	4.2318	**Er**	3.5229	3.021	**La**	8.5556	3.1377	**Ta**	40.765	2.8241
**Au**	19.6276	2.9337	**Es**	6.0393	2.9391	**Li**	12.5818	2.1836	**Tb**	3.5229	3.0745
**B**	90.5888	3.6375	**Eu**	4.0262	3.1119	**Lr**	5.536	2.8829	**Tc**	24.157	2.6709
**Ba**	183.1907	3.299	**F**	25.1636	2.997	**Lu**	20.6341	3.2429	**Te**	200.302	3.9823
**Be**	42.7781	2.4455	**Fe**	6.5425	2.5943	**Md**	5.536	2.9168	**No**	5.536	2.8936
**Bi**	260.6945	3.8932	**Fm**	6.0393	2.9275	**Mg**	55.8631	2.6914	**Np**	9.5622	3.0504
**Bk**	6.5425	2.9747	**Fr**	25.1636	4.3654	**Mn**	6.5425	2.638	**O**	30.1963	3.1181
**Br**	126.3211	3.732	**Ga**	208.8576	3.9048	**Mo**	28.1832	2.719	**Os**	18.621	2.7796
**C**	52.8435	3.4309	**Gd**	4.5294	3.0005	**N**	34.7257	3.2607	**Rh**	26.6734	2.6094
**Ca**	119.7786	3.0282	**Ge**	190.7398	3.813	**Na**	15.0981	2.6576	**Rn**	124.8113	4.2451
**Cd**	114.7458	2.5373	**H**	22.1439	2.5711	**Nb**	29.693	2.8197	**Ru**	28.1832	2.6397
**Ce**	6.5425	3.168	**He**	28.1832	2.1043	**Nd**	5.0327	3.185	**S**	137.8963	3.5948
**Cf**	6.5425	2.9515	**Hf**	36.2355	2.7983	**Ne**	21.1374	2.8892	**Sb**	225.9688	3.9378
**Cl**	114.2426	3.5164	**Hg**	193.7594	2.4099	**Ni**	7.5491	2.5248	**Y**	36.2355	2.9801
**P**	153.4977	3.6946	**Th**	13.0851	3.0255	**Pt**	40.2617	2.4535	**Yb**	114.7458	2.989
**Pa**	11.072	3.0504	**Ti**	8.5556	2.8286	**Pu**	8.0523	3.0504	**Zn**	62.4056	2.4616
**Pb**	333.6688	3.8282	**Tl**	342.2245	3.8727	**Ra**	203.3216	3.2758	**Zr**	34.7257	2.7832
**Pd**	24.157	2.5827	**Tm**	3.0196	3.0059	**Rb**	20.1309	3.6652	**Po**	163.5632	4.1952
**Pm**	4.5294	3.16	**U**	11.072	3.0246	**Re**	33.2159	2.6317	**Pr**	5.0327	3.2126
**Xe**	167.0861	3.9235	**V**	8.0523	2.801	**W**	33.7192	2.7342			
$\mathbf{C_{CO_2}}$	27.0	2.8	$\mathbf{O_{CO_2}}$	79.0	3.05	$\mathbf{CH_4}$	148.0	3.73			

Supplementary Note 12: Details of the Partial Charge Calculation using the EQeq method

The extended charge equilibration method[11] was used to compute the partial charges of the atoms in the frameworks of the MOFs. This method is the most suitable choice for the investigation of the tens of thousands of structures we considered in this study,[12] as DFT alternative methods, albeit more accurate, are prohibitive for this large number of structures. Moreover, this method can be applied to any MOF, being it consistently parametrized for all the elements up to Polonium. As for the selection of the reference charge centers, which informs the electronegativity and idempotential inputs for the program, we chose for each element the lowest common oxidation state: this protocol was used for the benchmark of EQeq in our previous work.[12] Oxidation states are listed in Table below. The values for the electronegativity and idempotential are computed from experimental data.[18] The code is available from the GitHub repository https://github.com/danieleongari/EQeq/releases/tag/v1.1.0, and the default settings from the release v1.1.0 have been used for all the calculations.

Supplementary Table 14. list of lowest common oxidation states selected as charge center for the EQeq calculations.

Atom type	Oxidation state	Atom type	Oxidation state	Atom type	Oxidation state	Atom type	Oxidation state	Atom type	Oxidation state	Atom type	Oxidation state
H	0	S	0	Ge	0	Ag	1	Sm	1	Ir	0
He	0	Cl	0	As	0	Cd	2	Eu	1	Pt	1
Li	1	Ar	0	Se	0	In	3	Gd	1	Au	1
Be	2	K	1	Br	0	Sn	2	Tb	1	Hg	1
B	0	Ca	2	Kr	0	Sb	0	Dy	1	Tl	1
C	0	Sc	3	Rb	1	Te	0	Ho	1	Pb	2
N	0	Ti	4	Sr	2	I	0	Er	1	Bi	3
O	0	V	4	Y	3	Xe	0	Tm	2	Po	0
F	0	Cr	3	Zr	4	Cs	1	Yb	2	Ga	3
Ne	0	Mn	2	Nb	4	Ba	1	Lu	1	P	0
Na	1	Fe	2	Mo	4	La	2	Hf	3	Zn	2
Mg	2	Co	2	Tc	2	Ce	3	Ta	0	Pd	2
Al	3	Ni	2	Ru	2	Pr	3	W	0	Pm	1
Si	0	Cu	2	Rh	2	Nd	1	Re	0	Os	0

Supplementary Note 13: Including hMOF in the Design Space

Here we show how **hMOF** is covering the design space. We included 10,000 structures sampled from this database using MaxMin method to the structures from **CoRE-2019**, **BW-DB**, **ToBaCCo**. In Supplementary Figure 21, we show the pore geometry maps for this more populated space. The difference between the shape of maps in this figure and the figures in the main text is due to the stochastic nature of t-SNE method. However, the same information is encoded in the figures, e.g., **CoRE-2019** is mainly in small pore regions and **ToBaCCo** is covering the large pore region. The distribution of the geometric features in **hMOF** is very similar to those from **BW-DB**.

In Supplementary Figure 22, we show the coverage of the three chemistry domains by **hMOF**. We see that this hypothetical database has similar distribution as **BW-DB**. Very little coverage of the metal chemistry and good coverage of functional groups and linker chemistry. The **hMOF** covers more in the metal chemistry map. This is simply an artefact as many structures are unphysical. These unphysical structures are present in the database since the structures were not geometry optimized and there are many clashing/close contacts atoms. Despite using only 5 metal centers, our unique graph identification method finds more than 1200 unique metal centers in this database. We show examples for these unphysical structures in Supplementary Figure 23 for each of the original metal centers.

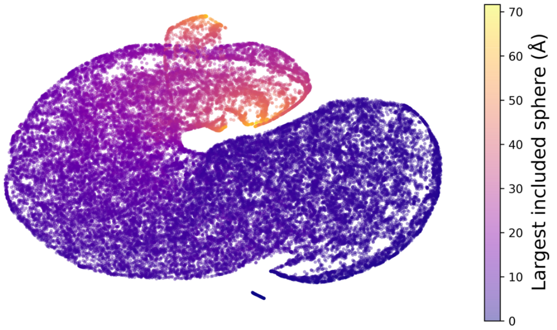

(a)

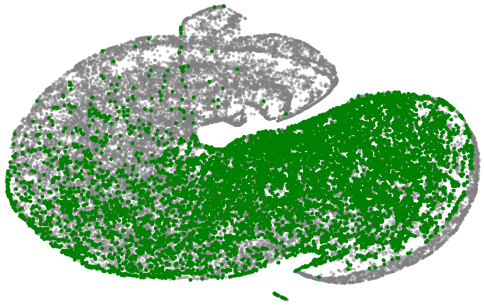

(b)

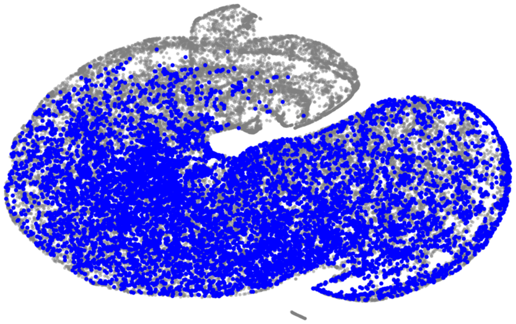

(c)

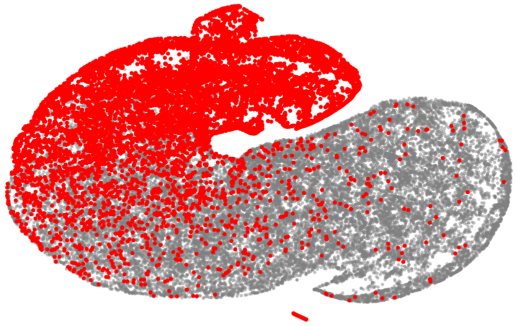

(d)

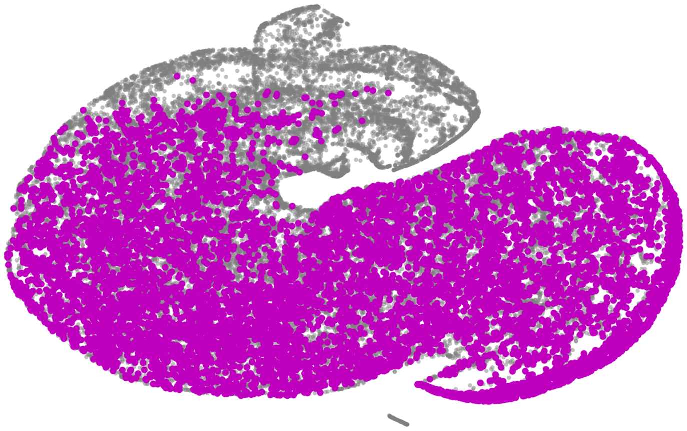

(e)

Supplementary Figure 21 The t-SNE representation of the geometric descriptors for the databases including **hMOF**. Top left figure is color coded with the largest included sphere. (b), (c), (d), and (e) show the distribution of **CoRE-2019**, **BW-DB**, **ToBaCCo**, and **hMOF**, respectively.

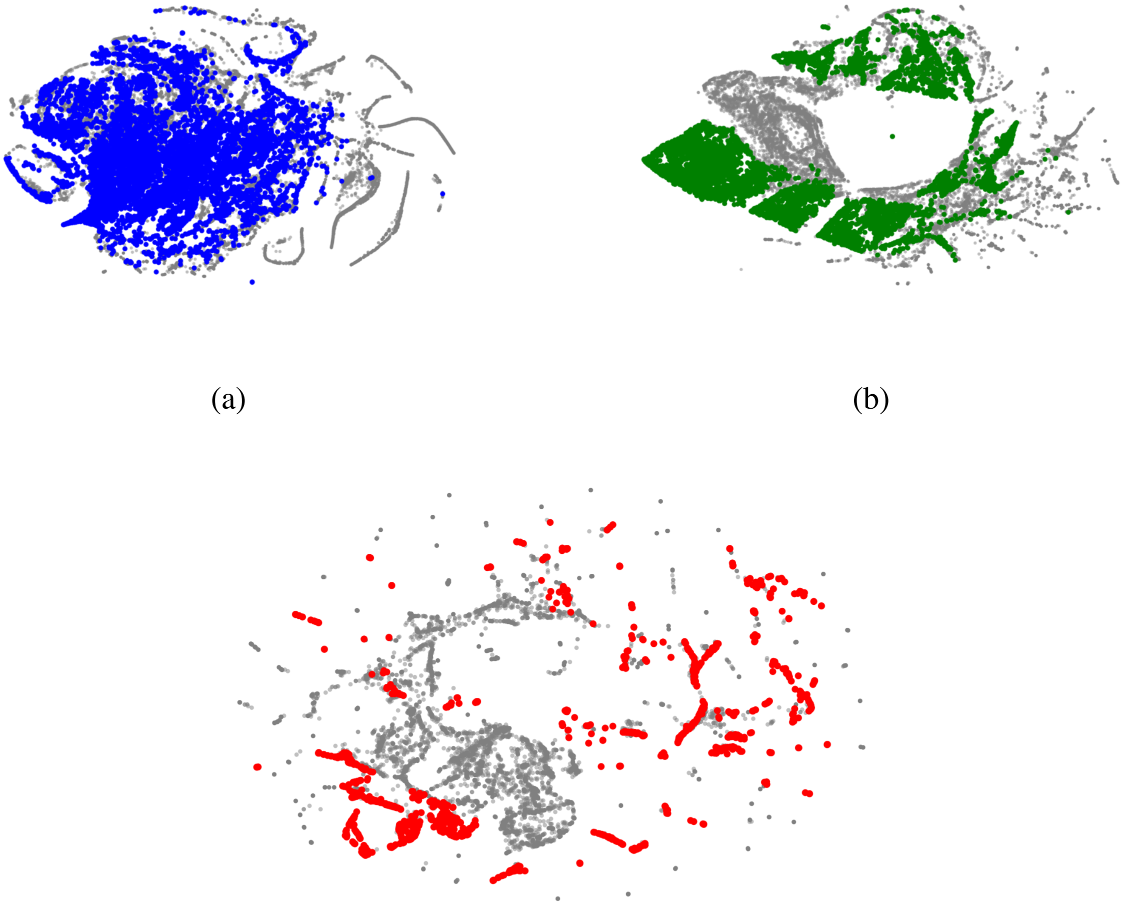

(a) (b) (c)

Supplementary Figure 22. The t-SNE representation of the coverage of the three different domains of MOF chemistry by **hMOF**: (a) linker chemistry, (b) functional groups, (c) metal centers. The large number of points from **hMOF** in the metal center map is an artifact of the unphysical chemistry in the database.

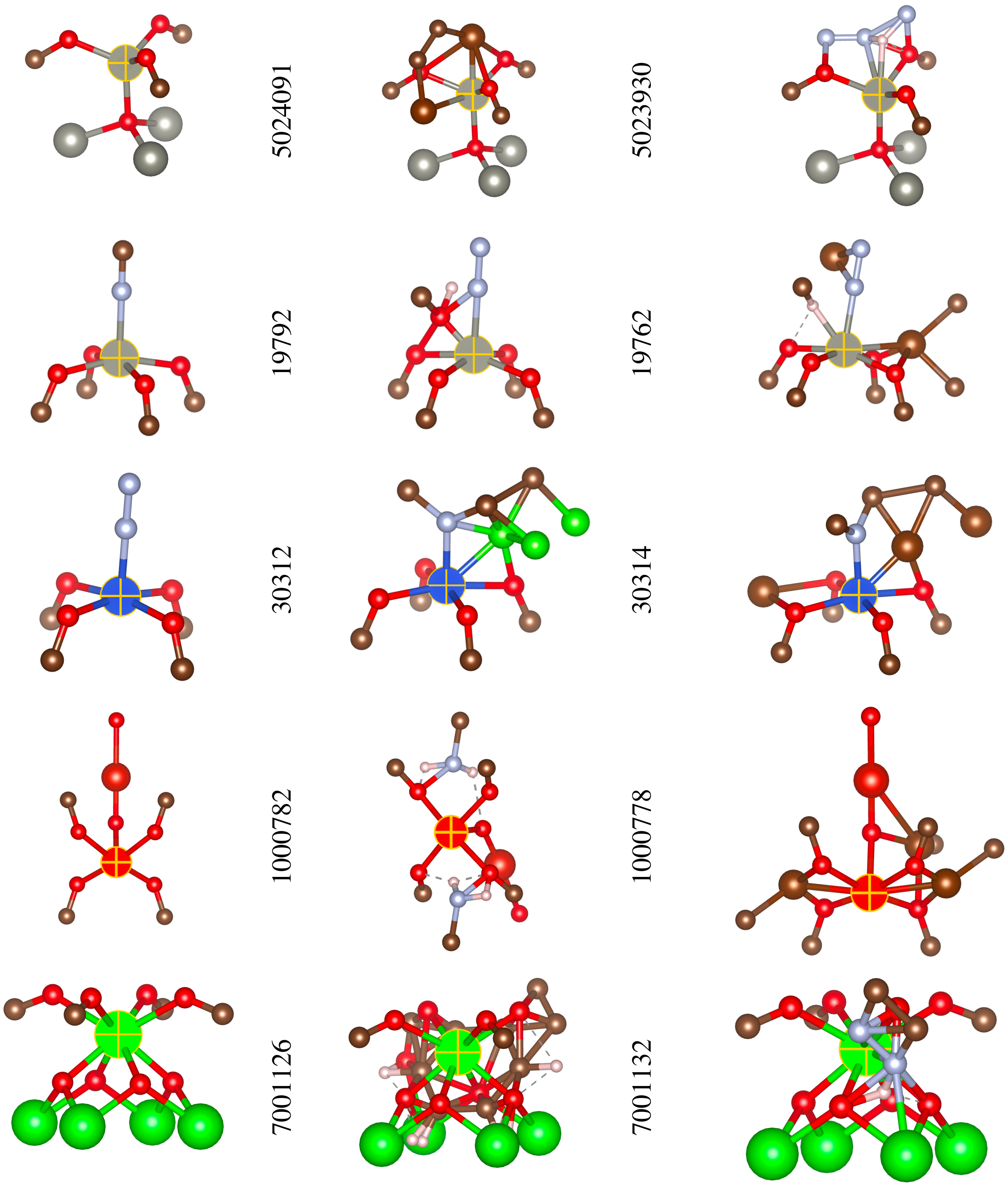

Supplementary Figure 23. Examples of unphysical structures in **hMOF** for each of the metal centers. The atoms up to the second coordination shell are shown in this figure. The number next to the representations correspond to the structure number in **hMOF**. The central metal of each complex is specified with a gold + sign.

Supplementary Note 14: Metal Type Variation in Databases

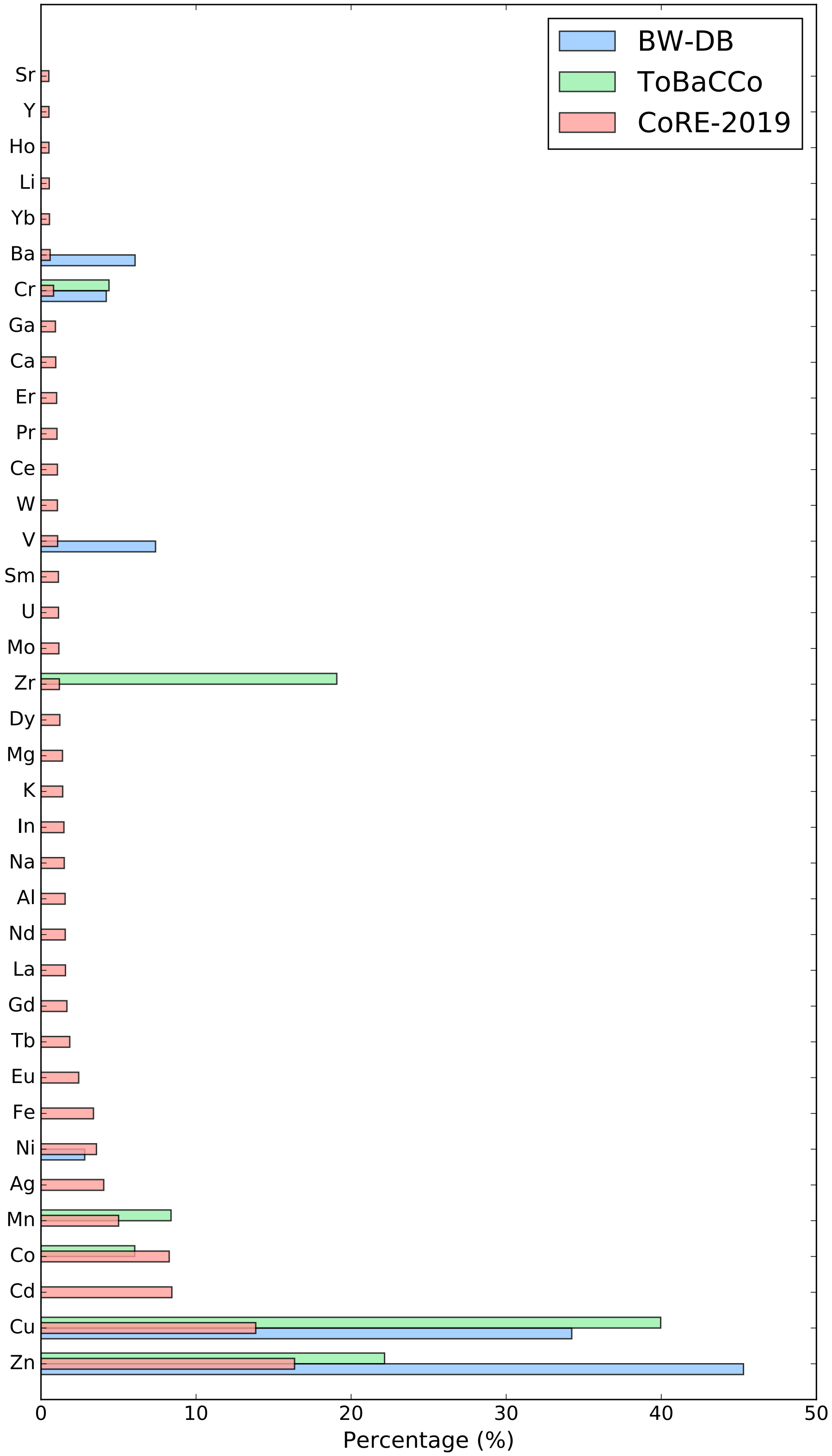

Supplementary Figure 24. The distribuion of the metal types in the MOF databases.

Supplementary Note 15: Distribution of Adsorption Properties of Databases

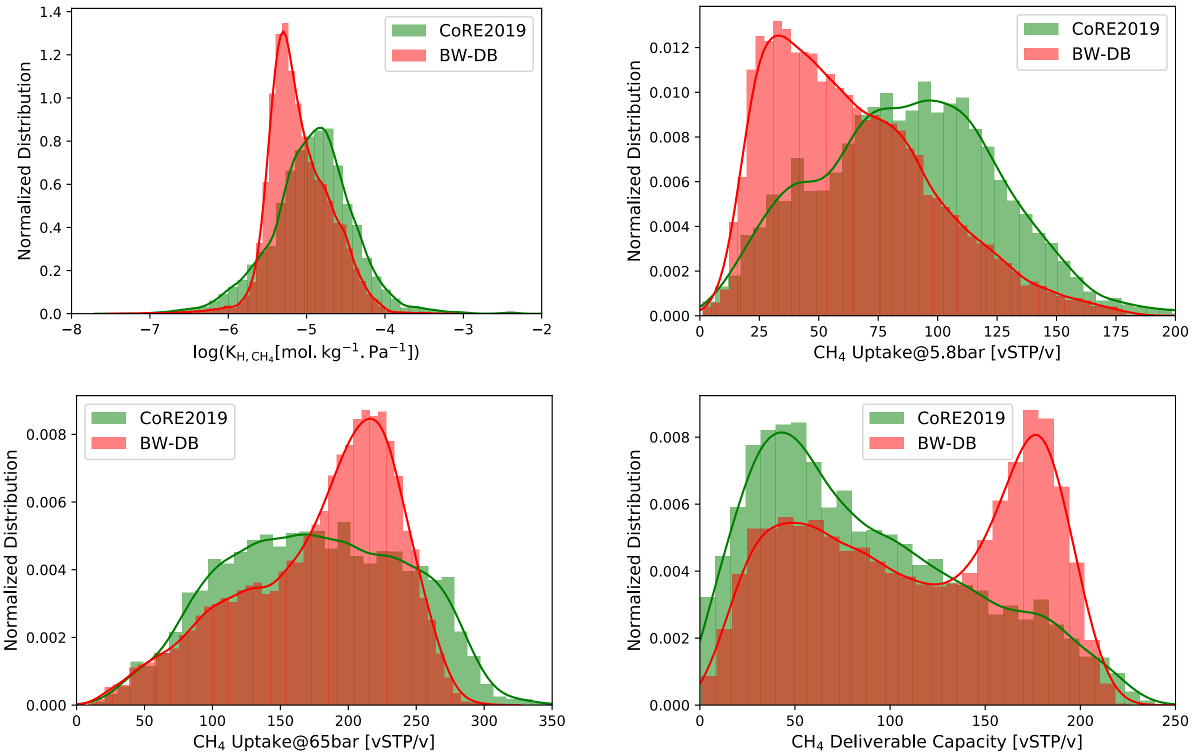

Supplementary Figure 25. Distribution of methane adsorption properties in BW-DB and CoRE-2019.

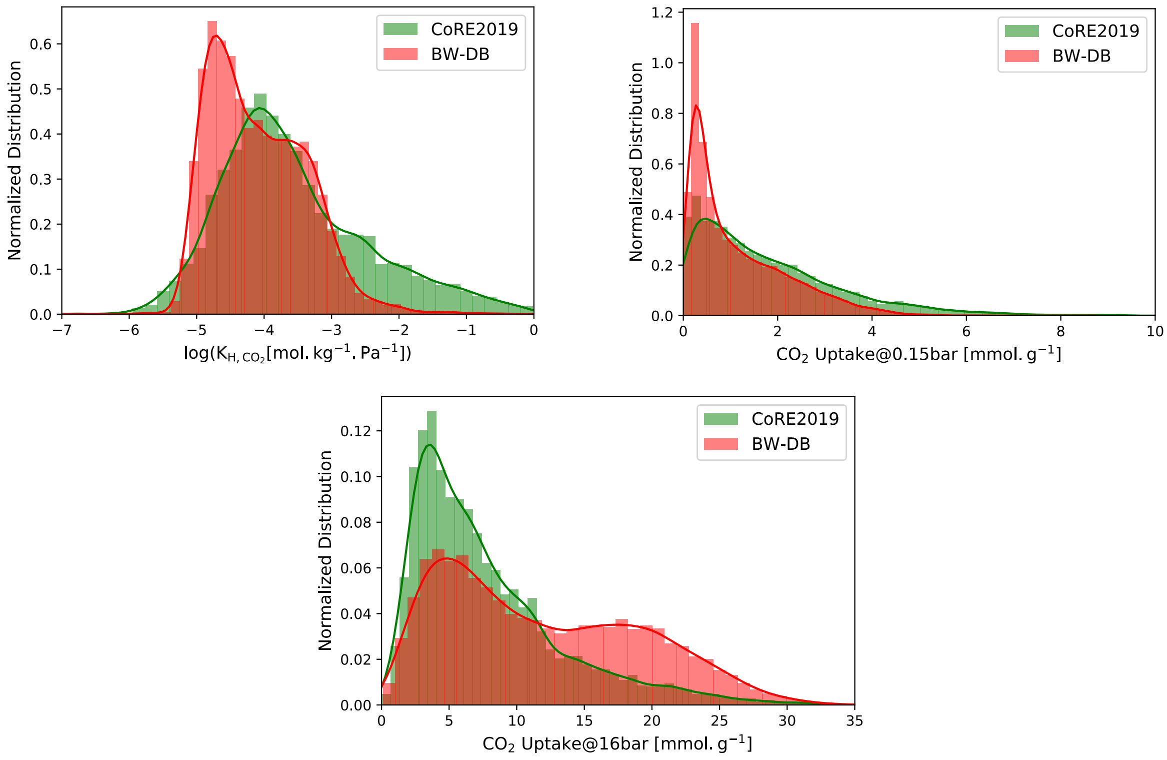

Supplementary Figure 26. Distribution of carbon dioxide adsorption properties in BW-DB and CoRE-2019.

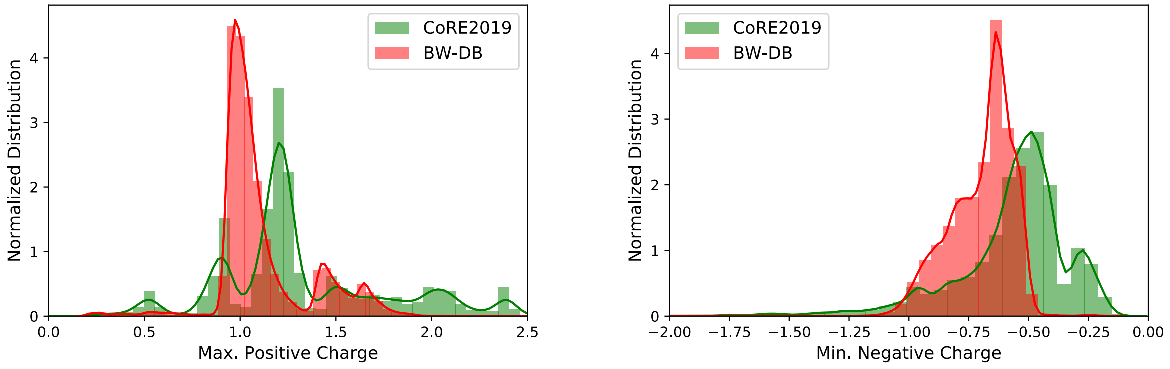

Supplementary Figure 27. Distribution of partial atomic charges in BW-DB and CoRE-2019.

Supplementary Note 16: Diversity Metrics

We describe diversity with three metrics: variety, balance, and disparity. For variety and balance, we first split the space to 1000 bins using k-means method. Variety is the number of sampled bins by each database, i.e., how many district types of structure exist in a database normalized with the 1000 unique bins. Balance shows how the evenness of the distribution of structures among the sample bins. Different methods exist for computing evenness which are all transformation of Shannon entropy. Shannon entropy measures the stochastic nature of data by computing the relative chance that a sample from a distribution would be from a given kind and it is computed as:

$$H(X) = -\sum P(\mathrm{x_i}) \log\, P(\mathrm{x_i}) \qquad \text{Supplementary Equation (S 3)}$$

Based on this equation, the maximum entropy would be achieved when all bins are equally likely, i.e., uniform distribution. Therefore, a metric for evenness is relative entropy that is normalizing the entropy of the system with the maximum entropy (the entropy of a uniform distribution):

$$H_{rel}(X) = \frac{\exp\,(H(X))}{\exp(\,H_{max})} \qquad \text{Supplementary Equation (S 4)}$$

Here, we normalize with exponential of max entropy distribution because this term becomes linear with respect to the size of system, i.e., number of bins. Another flavor of the relative entropy is Kullback-Leibler divergence which measures the difference between two probability distributions:

$$D_{KL}(P||Q) = \sum P(\mathrm{x_i}) \log \frac{P(x_i)}{Q(x_i)}. \qquad \text{Supplementary Equation (S 5)}$$

A nice behaving transformation of entropy was introduced by Pielou:

$$PL_{rel}(X) = \frac{1 - \exp\,(H(X))}{1 - \exp(\,H_{max})} \qquad \text{Supplementary Equation (S 6)}$$

In this work, we use $1 - PL_{rel}(X)$ to have a measure of evenness of distribution such that 1 is the maximum evenness, i.e., the uniform distribution.

In figures below, the distribution of structures among the sampled bins are shown.

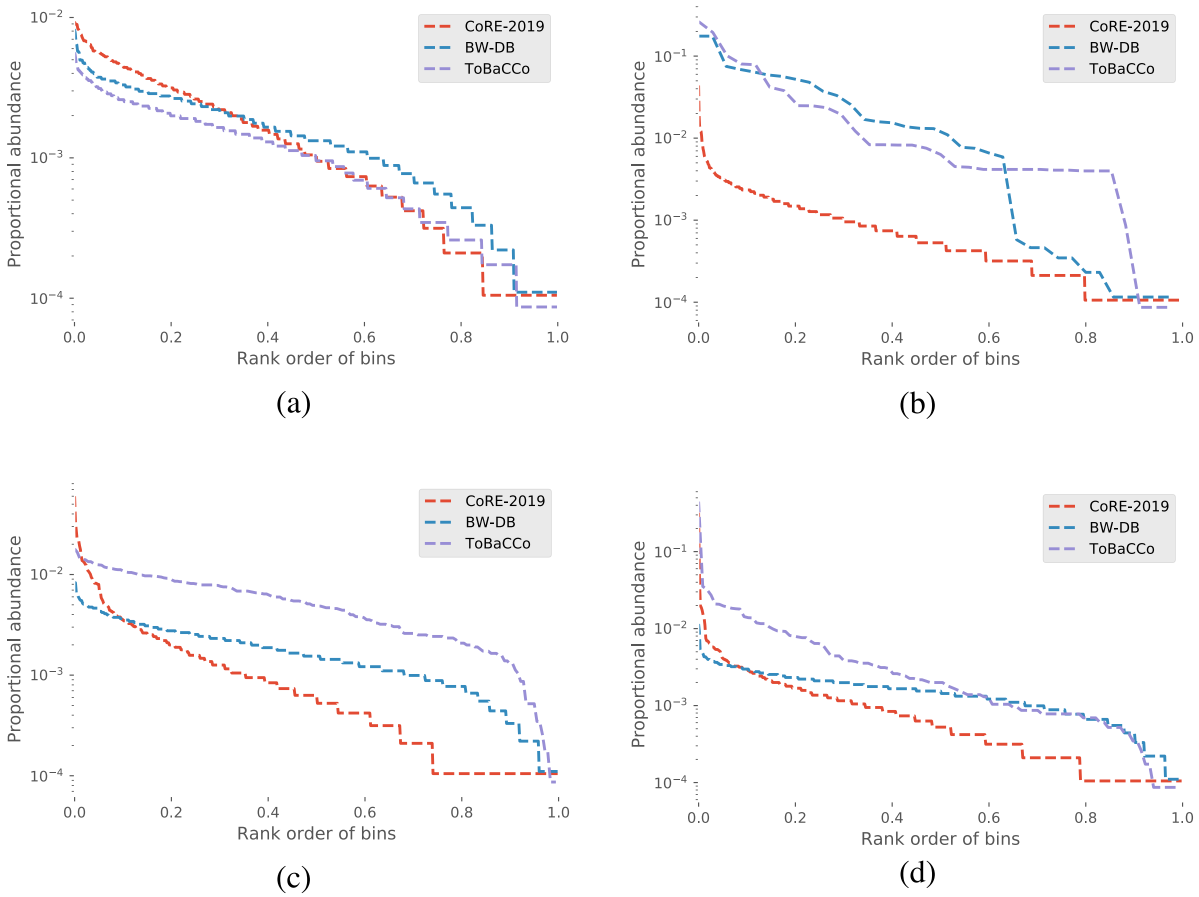

Supplementary Figure 28. Evenness of distribution of structures among the sampled bins for by each database. A flat distribution would lead to perfect evenness, i.e., Pielou's evenness factor of 1. (a) geometry, (b) metal chemistry, (c) linker chemistry, and (d) functional groups.

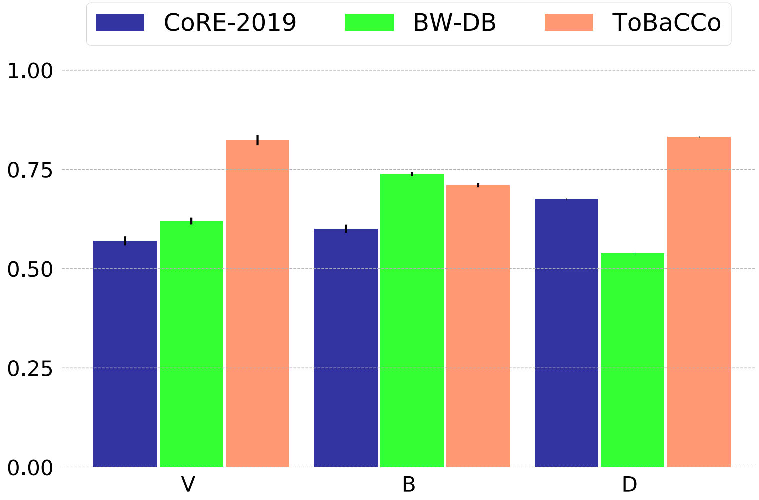

	Variety	KL	Relative Entropy	Pielou's Evenness	Disparity
**CoRE-2019**	0.57 (0. 011)	0.51 (0.017)	0.955 (0.0027)	0.60 (0.010)	0.68
**BW-DB**	0.62 (0.009)	0.30 (0.007)	0.974 (0.0011)	0.74 (0.005)	0.54
**ToBaCCo**	0.82 (0.013)	0.34 (0.008)	0.973 (0.0013)	0.71 (0.005)	0.83

Supplementary Figure 29. Diversity metrics for the geometric features of the three databases. The numbers in parantesis show the standard deviation of the metrics for changing the number of bins from 800 to 1200.

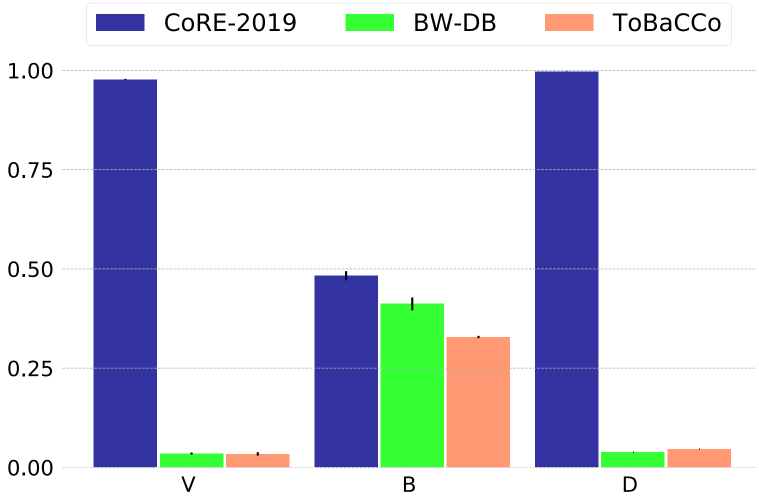

	Variety	KL	Relative Entropy	Pielou's Evenness	Disparity
**CoRE-2019**	0.977 (0.015)	0.72 (0.023)	0.942 (0.000)	0.48 (0.012)	0.997
**BW-DB**	0.035 (0.002)	0.85 (0.039)	0.785 (0.003)	0.41 (0.016)	0.039
**ToBaCCo**	0.034 (0.004)	1.05 (0.009)	0.717 (0.000)	0.32 (0.003)	0.046

Supplementary Figure 30. Diversity metrics for the metal center features of the three databases. The numbers in parantesis show the standard deviation of the metrics for changing the number of bins from 800 to 1200.

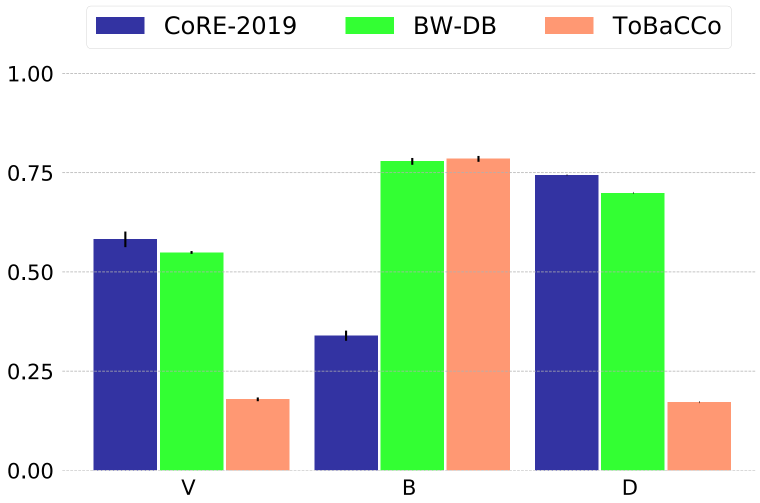

	Variety	KL	Relative Entropy	Pielou's Evenness	Disparity
**CoRE-2019**	0.58 (0.020)	1.08 (0.035)	0.900 (0.005)	0.34 (0.013)	0.74
**BW-DB**	0.55 (0.004)	0.25 (0.011)	0.978 (0.001)	0.78 (0.008)	0.70
**ToBaCCo**	0.18 (0.005)	0.24 (0.009)	0.971 (0.001)	0.78 (0.007)	0.17

Supplementary Figure 31. Diversity metrics for the linker chemistry features of the three databases. The numbers in parantesis show the standard deviation of the metrics for changing the number of bins from 800 to 1200.

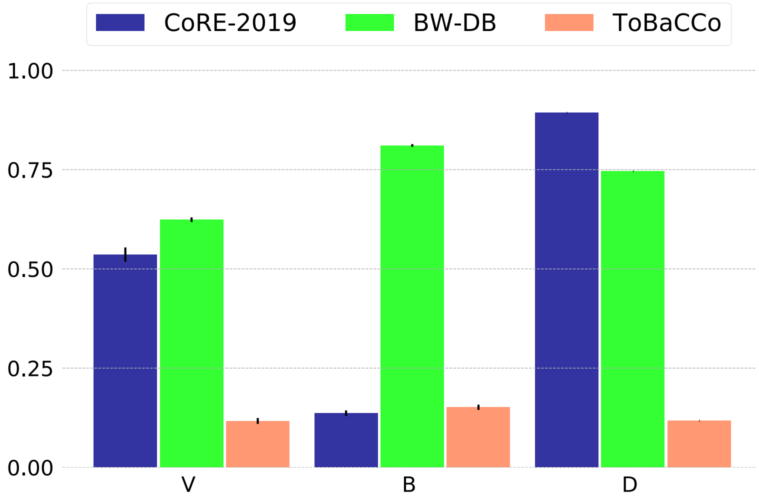

	Variety	KL	Relative Entropy	Pielou Evenness	Disparity
**CoRE-2019**	0.54 (0.018)	1.98 (0.050)	0.794 (0.002)	0.14 (0.007)	0.89
**BW-DB**	0.62 (0.006)	0.21 (0.004)	0.982 (0.001)	0.81 (0.003)	0.75
**ToBaCCo**	0.12 (0.007)	1.84 (0.043)	0.687 (0.004)	0.15 (0.007)	0.18

Supplementary Figure 32. Diversity metrics for the functional group features of the three databases. The numbers in parantesis show the standard deviation of the metrics for changing the number of bins from 800 to 1200.

Supplementary Note 17: Learning Curves for Different Train Sets

Using a diverse set for training ML models should in principle help us to have a more efficient learning. Here, we show this using learning curves of random forest (RF) and k-nearest-neighbors (k-NN) models. The k-NN model is particularly illustrative because this model only uses the k neighbors in feature space for predictions, and therefore, the distribution of the train set in the entire feature space would significantly influence the performance of this model. Figures below show that both RF and k-NN (n=5, using Euclidian distances) models trained using the diverse set are significantly more transferable to both databases. In contrast, the models trained on one database have very poor transferability to the other databases.

Moreover, the steepness and saturation point of the learning curve can provide an indication of minimum database size. For the properties that depend only on a few structural parameters, e.g., the high-pressure gas uptake that mainly rely on the pore volume, the learning curve is very steep and saturates very fast. For such properties, the number of structures that diversely sample the range of pore volumes is indeed smaller than for those properties that rely on many structural parameters, e.g., low-pressure CO_2 uptake that rely on both geometry and chemistry.

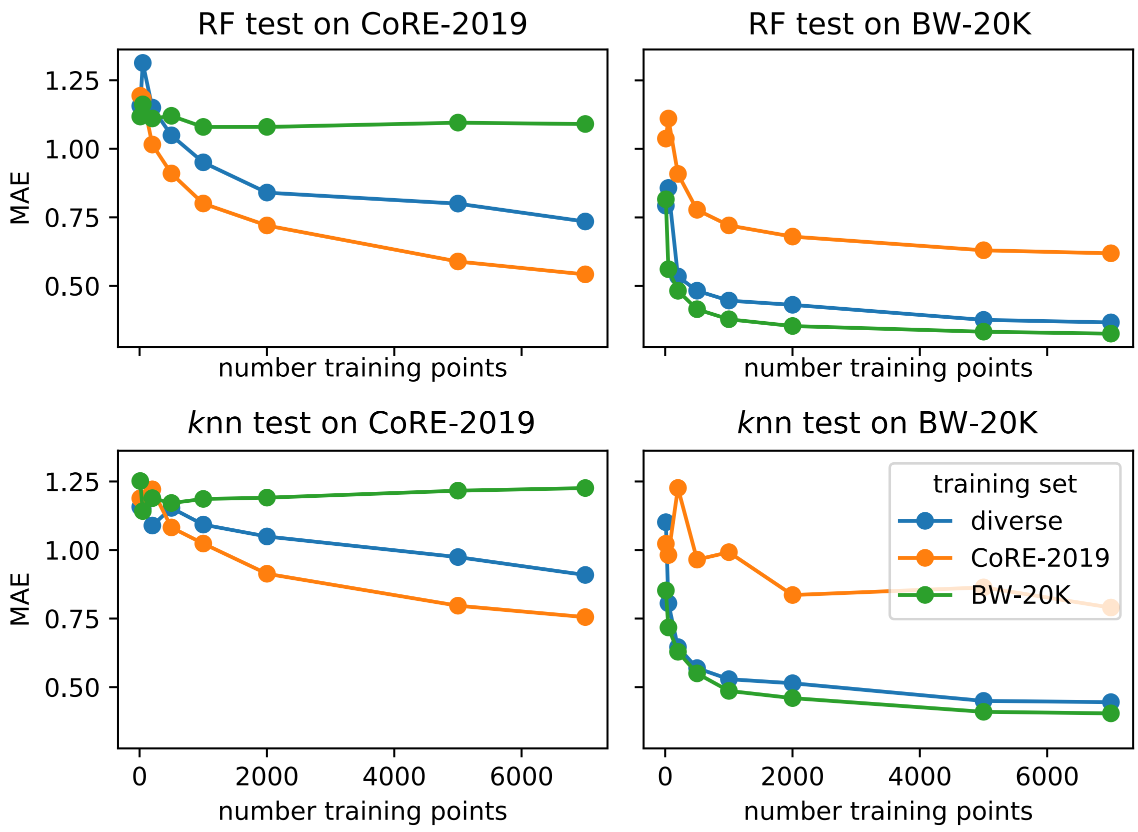

Supplementary Figure 33. Learning curves of RF and kNN models for predictions of CO_2 uptake at low pressures using different training sets.

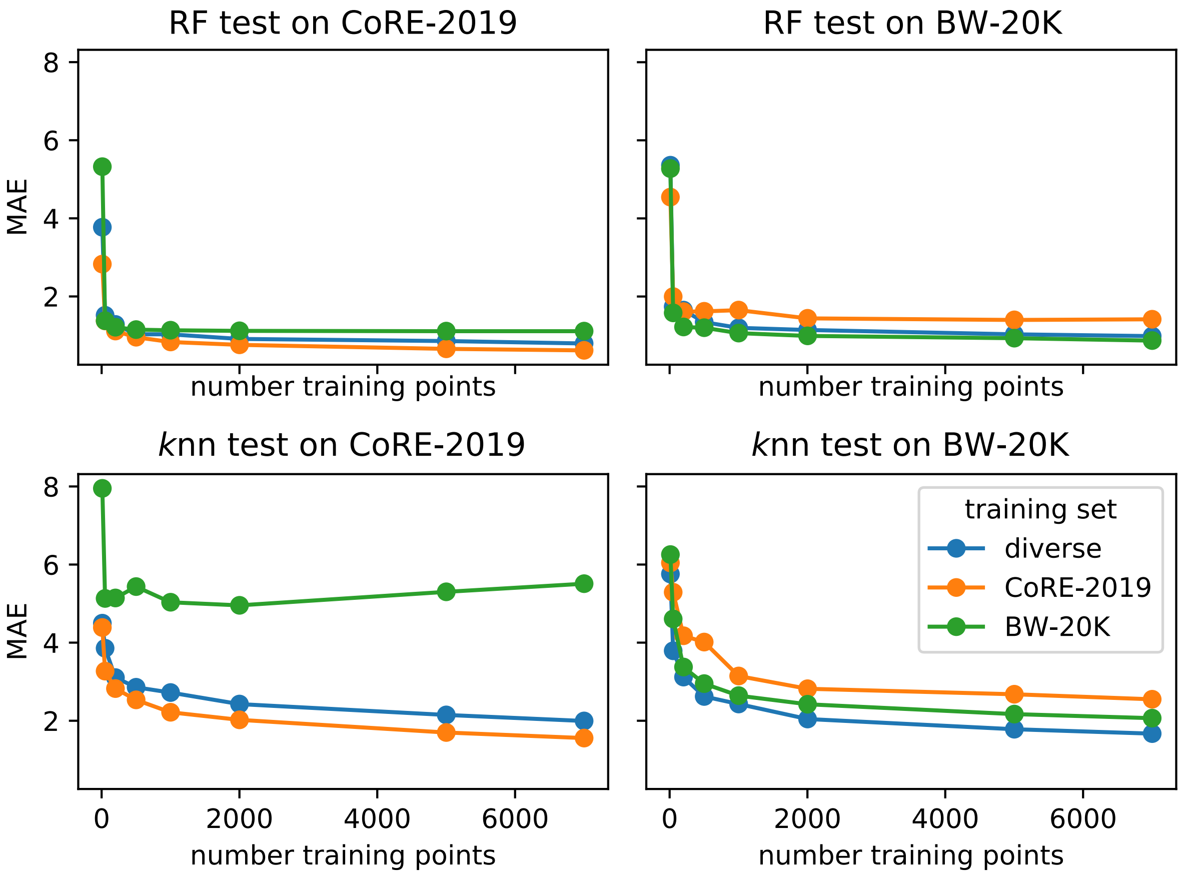

Supplementary Figure 34. Learning curves of RF and kNN models for predictions of CO_2 uptake at high pressures using different training sets.

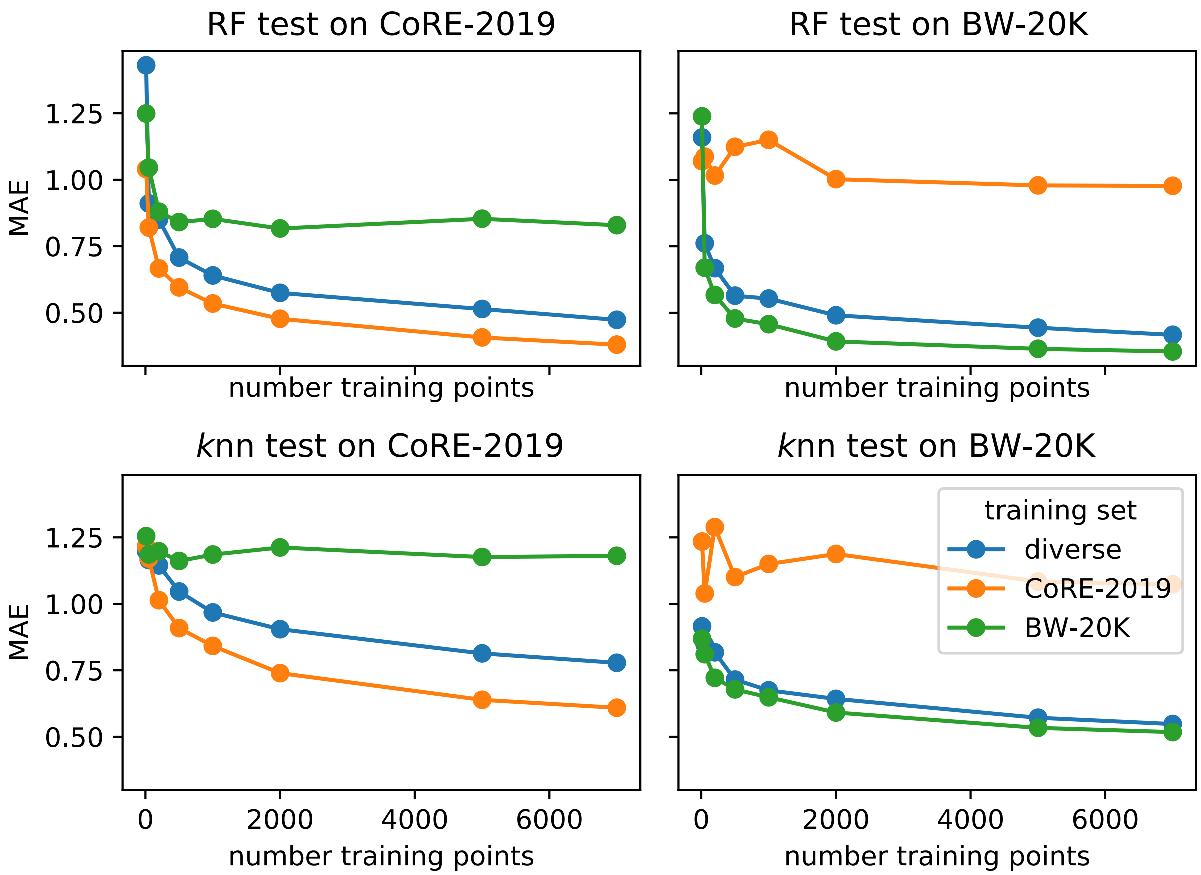

Supplementary Figure 35. Learning curves of RF and kNN models for predictions of methane uptake at low pressures using different training sets.

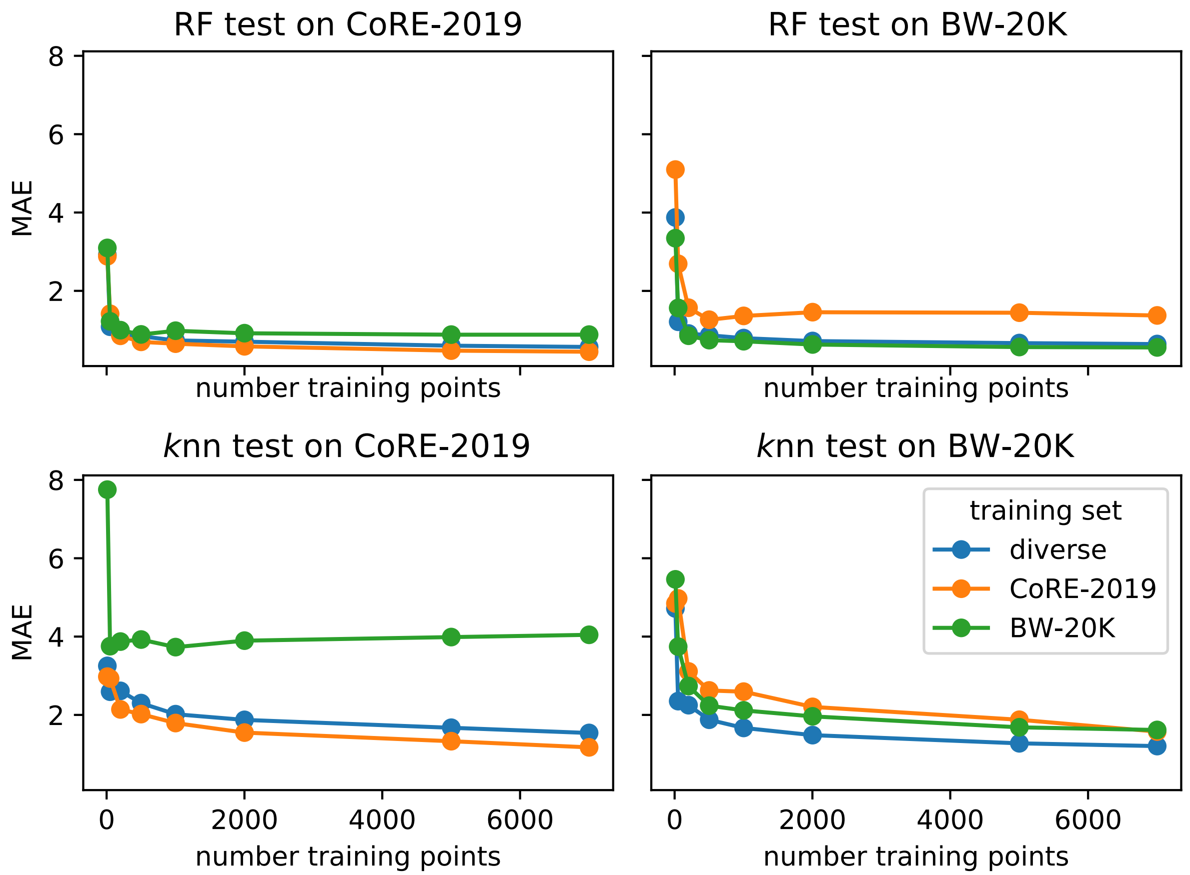

Supplementary Figure 36. Learning curves of RF and kNN models for predictions of methane uptake at high pressures using different training sets.

Supplementary References

(1) Ong, S. P.; Richards, W. D.; Jain, A.; Hautier, G.; Kocher, M.; Cholia, S.; Gunter, D.; Chevrier, V. L.; Persson, K. A.; Ceder, G. Python Materials Genomics (Pymatgen): A Robust, Open-Source Python Library for Materials Analysis. *Comput. Mater. Sci.* **2013**, *68*, 314–319.

(2) Boyd, P. G.; Moosavi, S. M.; Witman, M.; Smit, B. Force-Field Prediction of Materials Properties in Metal-Organic Frameworks. *J. Phys. Chem. Lett.* **2017**, *8* (2), 357–363.

(3) Ioannidis, E. I.; Gani, T. Z. H.; Kulik, H. J. MolSimplify: A Toolkit for Automating Discovery in Inorganic Chemistry. *J. Comput. Chem.* **2016**, *37* (22), 2106–2117.

(4) Chung, Y. G.; Camp, J.; Haranczyk, M.; Sikora, B. J.; Bury, W.; Krungleviciute, V.; Yildirim, T.; Farha, O. K.; Sholl, D. S.; Snurr, R. Q. Computation-Ready, Experimental Metal--Organic Frameworks: A Tool to Enable High-Throughput Screening of Nanoporous Crystals. *Chem. Mater.* **2014**, *26* (21), 6185–6192.

(5) Chung, Y. G.; Haldoupis, E.; Bucior, B. J.; Haranczyk, M.; Lee, S.; Zhang, H.; Vogiatzis, K. D.; Milisavljevic, M.; Ling, S.; Camp, J. S.; et al. Advances, Updates, and Analytics for the Computation-Ready, Experimental Metal--Organic Framework Database: CoRE MOF 2019. *J. Chem. Eng. Data* **2019**, *64* (12), 5985–5998.

(6) Nazarian, D.; Camp, J. S.; Sholl, D. S. A Comprehensive Set of High-Quality Point Charges for Simulations of Metal--Organic Frameworks. *Chem. Mater.* **2016**, *28* (3), 785–793.

(7) Wilmer, C. E.; Leaf, M.; Lee, C. Y.; Farha, O. K.; Hauser, B. G.; Hupp, J. T.; Snurr, R. Q. Large-Scale Screening of Hypothetical Metal--Organic Frameworks. *Nat. Chem.* **2012**, *4* (2), 83.

(8) Boyd, P. G.; Woo, T. K. A Generalized Method for Constructing Hypothetical Nanoporous Materials of Any Net Topology from Graph Theory. *CrystEngComm* **2016**, *18* (21), 3777–3792.

(9) Gómez-Gualdrón, D. A.; Colón, Y. J.; Zhang, X.; Wang, T. C.; Chen, Y.-S.; Hupp, J. T.; Yildirim, T.; Farha, O. K.; Zhang, J.; Snurr, R. Q. Evaluating Topologically Diverse Metal--Organic Frameworks for Cryo-Adsorbed Hydrogen Storage. *Energy Environ. Sci.* **2016**, *9* (10), 3279–3289.

(10) Anderson, R.; Rodgers, J.; Argueta, E.; Biong, A.; Gomez-Gualdron, D. A. Role of Pore Chemistry and Topology in the CO2 Capture Capabilities of MOFs: From Molecular Simulation to Machine Learning. *Chem. Mater.* **2018**, *30* (18), 6325–6337.

(11) Wilmer, C. E.; Kim, K. C.; Snurr, R. Q. An Extended Charge Equilibration Method. *J. Phys. Chem. Lett.* **2012**, *3* (17), 2506–2511.

(12) Ongari, D.; Boyd, P. G.; Kadioglu, O.; Mace, A. K.; Keskin, S.; Smit, B. Evaluating Charge Equilibration Methods to Generate Electrostatic Fields in Nanoporous Materials. *J. Chem. Theory Comput.* **2018**, *15* (1), 382–401.

(13) Nandy, A.; Duan, C.; Janet, J. P.; Gugler, S.; Kulik, H. J. Strategies and Software for Machine Learning Accelerated Discovery in Transition Metal Chemistry. *Ind. Eng. Chem. Res.* **2018**, *57* (42), 13973–13986.

(14) Pedregosa, F.; Varoquaux, G.; Gramfort, A.; Michel, V.; Thirion, B.; Grisel, O.; Blondel, M.; Prettenhofer, P.; Weiss, R.; Dubourg, V.; et al. Scikit-Learn: Machine Learning in

Python. *J. Mach. Learn. Res.* **2011**, *12* (Oct), 2825–2830.

(15) Strobl, C.; Boulesteix, A.-L.; Zeileis, A.; Hothorn, T. Bias in Random Forest Variable Importance Measures: Illustrations, Sources and a Solution. *BMC Bioinformatics* **2007**, *8* (1), 25.

(16) Rappé, A. K.; Casewit, C. J.; Colwell, K. S.; Goddard III, W. A.; Skiff, W. M. UFF, a Full Periodic Table Force Field for Molecular Mechanics and Molecular Dynamics Simulations. *J. Am. Chem. Soc.* **1992**, *114* (25), 10024–10035.

(17) Martin, M. G.; Siepmann, J. I. Transferable Potentials for Phase Equilibria. 1. United-Atom Description of n-Alkanes. *J. Phys. Chem. B* **1998**, *102* (14), 2569–2577.

(18) Moore, C. E. *Ionization Potentials and Ionization Limits Derived from the Analyses of Optical Spectra*; 1970.
